# PET imaging of microglia in Alzheimer's disease using copper-64 labeled TREM2 antibodies

**DOI:** 10.7150/thno.97149

**Published:** 2024-09-30

**Authors:** Monireh Shojaei, Rebecca Schaefer, Kai Schlepckow, Lea H. Kunze, Felix L. Struebing, Bettina Brunner, Michael Willem, Laura M. Bartos, Astrid Feiten, Giovanna Palumbo, Thomas Arzberger, Peter Bartenstein, Gian Carlo Parico, Dan Xia, Kathryn M. Monroe, Christian Haass, Matthias Brendel, Simon Lindner

**Affiliations:** 1 Department of Nuclear Medicine, University Hospital, LMU Munich, Munich, Germany.; 2 German Center for Neurodegenerative Diseases (DZNE), Munich, Germany.; 3 Center for Neuropathology and Prion Research, University Hospital, LMU Munich, Munich, Germany.; 4 Metabolic Biochemistry, Biomedical Center (BMC), Faculty of Medicine, LMU Munich, Munich, Germany.; 5 Department of Psychiatry and Psychotherapy, University Hospital, LMU Munich, Munich, Germany.; 6 Munich Cluster of Systems Neurology (SyNergy), Munich, Germany.; 7 Denali Therapeutics Inc, South San Francisco, CA, USA.

**Keywords:** TREM2, ATV:4D9, copper-64, microglia, PET

## Abstract

Triggering receptor expressed on myeloid cells 2 (TREM2) plays an essential role in microglia activation and is being investigated as a potential therapeutic target for modulation of microglia in several neurological diseases. In this study, we present the development and preclinical evaluation of ^64^Cu-labeled antibody-based PET radiotracers as tools for non-invasive assessment of TREM2 expression. Furthermore, we tested the potential of an antibody transport vehicle (ATV) that binds human transferrin receptor to facilitate transcytosis of TREM2 antibody-based radiotracers to the CNS and improve target engagement.

**Methods:** A TREM2 antibody with an engineered transport vehicle (ATV:4D9) and without (4D9) were covalently modified with *p*NCS-benzyl-NODAGA and labeled with copper-64. Potency, stability, and specificity were assessed *in vitro* followed by *in vivo* PET imaging at the early 2 h, intermediate 20 h, and late imaging time points 40 h post-injection using a human transferrin receptor (hTfR) expressing model for amyloidogenesis (5xFAD;TfR^mu/hu^) or wild-type mice (WT;TfR^mu/hu^), and hTfR negative controls. Organs of interest were isolated to determine biodistribution by *ex vivo* autoradiography. Cell sorting after *in vivo* tracer injection was used to demonstrate cellular specificity for microglia and to validate TREM2 PET results in an independent mouse model for amyloidogenesis (App^SAA^;TfR^mu/hu^). For translation to human imaging, a human TREM2 antibody (14D3) was radiolabeled and used for *in vitro* autoradiography on human brain sections.

**Results:** The ^64^Cu-labeled antibodies were obtained in high radiochemical purity (RCP), radiochemical yield (RCY), and specific activity. Antibody modification did not impact TREM2 binding. ATV:4D9 binding proved to be specific, and the tracer stability was maintained over 48 h. The uptake of [^64^Cu]Cu-NODAGA-ATV:4D9 in the brains of hTfR expressing mice was up to 4.6-fold higher than [^64^Cu]Cu-NODAGA-4D9 in mice without hTfR. TREM2 PET revealed elevated uptake in the cortex of 5xFAD mice compared to wild-type, which was validated by autoradiography. PET-to-biodistribution correlation revealed that elevated radiotracer uptake in brains of 5xFAD;TfR^mu/hu^ mice was driven by microglia-rich cortical and hippocampal brain regions. Radiolabeled ATV:4D9 was selectively enriched in microglia and cellular uptake explained PET signal enhancement in App^SAA^;TfR^mu/hu^ mice. Human autoradiography showed elevated TREM2 tracer binding in the cortex of patients with Alzheimer's disease.

**Conclusion:** [^64^Cu]Cu-NODAGA-ATV:4D9 has potential for non-invasive assessment of TREM2 as a surrogate marker for microglia activation *in vivo*. ATV engineering for hTfR binding and transcytosis overcomes the blood-brain barrier restriction for antibody-based PET radiotracers. TREM2 PET might be a versatile tool for many applications beyond Alzheimer's disease, such as glioma and chronic inflammatory diseases.

## Introduction

Neurodegenerative diseases (NDD) are characterized by the gradual degeneration and loss of neurons within the central nervous system (CNS) 1, 2. This neuronal damage leads to cognitive and movement dysfunctions observed in diseases like Alzheimer's (AD) and Parkinson's disease (PD). NDDs are also associated with the accumulation and misfolding of amyloidogenic proteins 3, 4. In addition, neuroinflammation is observed in many NDDs and chronic inflammation contributes significantly to the development and pathology of the disease 5, 6.

Microglia, the main resident macrophages in the CNS, exhibit different phenotypes depending on the specific conditions of the brain microenvironment 6. In the healthy brain, resting microglia persist in a homeostatic state and play a crucial role in immune surveillance and maintaining brain homeostasis 7, 8. Upon a challenge such as accumulating amyloid pathology, microglia activate a defensive gene network 9, 10. Microglia that respond to pathological challenges, have a common transcriptional signature and are referred to as disease-associated microglia (DAM) 7, 11-13. Under persistent inflammatory conditions, microglia can remain activated losing their normal protective function and mediate chronic inflammatory processes 13-15. The dysregulation of the immune defense in the CNS seems to be an important factor in NDD progression and particularly emerges at an early stage in TREM2 late-onset Alzheimer's disease variants, even before other NDD pathologies come into play 8, 9, 16. Early diagnosis of NDDs is of utmost importance for patients as more therapy options can be applied 16. In this respect, positron emission tomography (PET) is an ideal diagnostic tool as it is a non-invasive imaging technology with high sensitivity and allows for accurate target detection and quantification at spatial resolution. Further, regional analyses of the entire brain by PET can identify heterogeneous target profiles and can detect regional changes by longitudinal imaging schemes.

The most investigated PET biomarker for activated microglia in the brain and spinal cord is the 18 kDa translocator protein (TSPO). TSPO is associated with the activation of microglia and is regarded as a distinctive characteristic of neuroinflammation. The initial tracer developed to target TSPO, (*R*)-[^11^C]PK11195, had certain drawbacks such as challenging radiochemistry, a short half-life and a low signal-to-noise ratio 17, 18. Among others, [^18^F]GE-180 is a widely used third-generation TSPO tracer with longer half-life, improved brain uptake and higher affinity 19. Although TSPO imaging provides useful clinical information, several drawbacks remain. Allelic variants in human populations with low TSPO binding hamper a broader use of TSPO PET for *in vivo* diagnostics 20. Furthermore, TSPO PET signals are not entirely specific to microglia, as TSPO is also expressed by endothelial cells, astrocytes and neurons 21. Limitations of TSPO as an imaging approach for microglia led us to investigate other biomarkers that are more microglia-specific, such as Triggering receptor expressed on myeloid cells 2 (TREM2) 16, 22, 23.

TREM2 is a type-1 transmembrane protein that plays an essential role in microglial functions 24, 25 and their transition from a homeostatic state to DAM 7, 11, 26, 27. Microglia combat amyloid pathology by phagocytosis and encapsulation of amyloid plaques 11, 24, 28, 29. TREM2 polymorphism, which has been reported previously as risk factor for late-onset AD (LOAD) 30, 31, impairs microglial activation leading to reduced Aβ plaque clearance 24, 32. Therapeutic approaches targeting TREM2 largely focus on agonistic antibodies stimulating protective microglial functions in the brain during the preclinical stage of AD 33. However, methods to assess direct target engagement are not available yet, and utilize an indirect measure of binding to soluble TREM2 present in biofluids 12. TSPO PET is not a suitable biomarker as it does not selectively represent TREM2-activated microglia. Thus, TREM2 PET would allow regional assessment of the TREM2-positive DAM subset and could be used as a novel biomarker to directly monitor CNS target engagement in preclinical studies and clinical trials.

Previous attempts to image TREM2 highlight the challenges in the CNS. In contrast, in the periphery, Shi *et al*. reported ^68^Ga-labeled COG1410, an ApoE-derived peptide that targets TREM2 on tumor-associated macrophages (TAMs) for the diagnosis of gastrointestinal tumors 34, and ^124^I- and ^99m^Tc-labeled antibody fragments showing potential for targeting human TREM2 in gastric carcinoma 35 and lung cancer 36. For neuroimaging, Meier *et al.* presented a ^124^I-labeled TREM2 antibody with a linked single-chain variable fragment that binds transferrin receptor to target TREM2 in AD mice. Using PET, the tracer was unable to image TREM2 *in vivo*, but could visualize TREM2* ex vivo* 8.

In our study, we focused on the development of a TREM2-targeting PET imaging agent based on the monoclonal agonistic antibody 4D9 that binds to the mouse TREM2 stalk region, *N*-terminal to the cleavage site of TREM2 12, and competes with α-secretase**-**mediated shedding and subsequent release of soluble TREM2 (sTREM2) 12, 37-40. TREM2 agonism is mediated by: 1) TREM2 stabilization at the cell surface by lowering TREM2 shedding; 2) Activation of phospho-SYK signaling downstream of TREM2 by antibody-mediated receptor cross-linking. These mechanisms lead to an increase in phagocytic uptake of Aβ fibrils by primary mouse microglia *in vitro* 12. To overcome the blood-brain barrier (BBB), which effectively restricts the delivery of antibodies into the brain, we engineered an ATV-enabled version of the mouse 4D9 antibody for use in our studies 41. The ATV selectively binds to the apical domain of the human transferrin receptor (hTfR) and facilitates active transport of the antibody into the brain parenchyma via TfR-mediated transcytosis within endothelial cells 41, 42.

We developed and evaluated two new radiotracers targeting TREM2, [^64^Cu]Cu‑NODAGA-ATV:4D9 and [^64^Cu]Cu‑NODAGA-4D9, i.e. the antibody chemically modified with the chelator NODAGA, which forms a complex with the radioisotope copper-64. These tracers enabled us to image TREM2 in the CNS of an AD mouse model. The effectiveness of tracer delivery into the brain was strongly enhanced by the ATV technology. Cell sorting after *in vivo* radiotracer injection proved microglia-specific binding of ATV:4D9.

## Materials and Methods

Detailed information on materials and experimental procedures are provided in the supplemental materials, including patient tissue, chemicals, p-SYK assay, arsenazo spectrophotometric assay, HPLC and TLC chromatograms, stability measurements, biodistribution data, PET images, Cohen's d calculations, tracer uptake data from regression analysis and flow cytometry data.

### Animals

All animal studies were carried out according to the German animal protection regulations and protocols, and a veterinarian was in charge of them (ROB-55.2-2532.Vet_02-21-156; ROB-55.2-2532.Vet_02-19-26). The animals were obtained from The Jackson Laboratory, Sacramento, CA, United States and Charles River, Sulzfeld, Germany. Mice were housed in isolated ventilated cages (IVC) at 23-26 °C and humidity levels of 55-60%, including a 12-hour light/dark cycle. Prior to the study, all mice were given at least two weeks of care and acclimatization. Following mouse cohorts were selected:

1A) 5xFAD (B6.Cg-Tg(APPSwFlLon,PSEN1*M146L*L286V)6799Vas/Mmjax), (n = 18, female = 9, male = 9)

1B) C57BL/6J, hereafter referred to as wild-type (WT) mice (n = 18, female = 8, male = 10)

2A) 5xFAD;TfR^mu/hu^ (hemizygous for 5xFAD, homozygous for TfR^mu/hu^), (n = 18, female = 9, male = 9)

2B) WT;TfR^mu/hu^ (homozygous for TfR^mu/hu^), (n = 18, female = 9, male = 9)

3) App^SAA^;TfR^mu/hu^ (homozygous for App^SAA^, homozygous for TfR^mu/hu^), (n = 4)

Mice from cohorts 2A, 2B and 3 express human transferrin receptors, with the apical domain of human TfR knocked into the mouse TfR locus. This approach preserves the native murine transferrin binding domain and retains the majority of the transferrin receptor gene 42. Mice from cohort 1 and 2 were age-matched (6-7 months) and included males and females. Cohort 3 mice (all male) were 19.7 ± 1.1 months old to ensure high TREM2 expression levels.

### Antibody modification

4D9, ATV:4D9 and ATV:ISO conjugation to the chelator 2,2′-(7-(1-carboxy-4-((4-isothiocyanatobenzyl)amino)-4-oxobutyl)-1,4,7-triazonane-1,4-diyl)diacetic acid (p-NCS-benzyl-NODAGA) was done as follows:

3.8 mg (7.3 µmol) *p*-NCS-benzyl-NODAGA was dissolved in a metal-free phosphate buffer (0.1 M, 100 µL, pH 8.5) and added to the antibody mixture (4 mg antibody in 1 mL PBS buffer). After the incubation of the reaction mixture (overnight, 4 °C) 43, the mixture was purified by Microcon® centrifugal filter units (Ultracel® 30 kDa, 0.5 mL, Merck Millipore Ltd). The HPLC (Agilent Technologies, 1200 series) quality control was done using a Phenomenex column (BioSep TM 5 m SEC s 4000 500 Å LC Column 300 × 7.8 mm) and sodium phosphate buffer (0.1 M, pH 7.2, 1 mL/min) as eluent. NODAGA-4D9 and bispecific NODAGA-ATV:4D9 antibodies revealed a retention time of 10.3 min and 10.4 min respectively in the UV channel at 280 nm. The number of chelators per antibody was determined using a spectrometric arsenazo assay as reported elsewhere 44.

### Radiolabeling

NODAGA-4D9, NODAGA-ATV:4D9 or NODAGA-ATV:ISO antibodies (100-200 µg) were incubated with 100-200 MBq of [^64^Cu]CuCl_2_ in a metal-free ammonium acetate buffer (0.1 M, pH 5.6, 42 °C). After the reaction (30 min), the buffer was changed to phosphate-buffered saline (ABX) via ultrafiltration (14000 g, 4 °C). Quality control was performed by radio-TLC (ITLC‑SG glass microfiber chromatography paper, Agilent Technologies, Folsom, CA) and radio-HPLC (BioSep TM 5 m SEC s 4000 500 Å LC column 300 × 7.8 mm, 0.1 M sodium phosphate buffer, pH 7.2, 1 mL/min). The retention factors in the TLC analysis were R_f_ ([^64^Cu]Cu‑NODAGA-4D9) = 0.0, R_f_ ([^64^Cu]Cu‑NODAGA-ATV:4D9) = 0.0, R_f_ ([^64^Cu]CuCl_2_) = 1.0, R_f_ ([^64^Cu]Cu‑NODAGA) = 0.5. [^64^Cu]Cu‑NODAGA-4D9 and [^64^Cu]Cu‑NODAGA-ATV:4D9 revealed a retention time of 10.3 and 10.7 minutes, respectively, by radio-HPLC.

### Autoradiography

Brain sections of 5xFAD;TfR^mu/hu^ and WT;TfR^mu/hu^ mice were pretreated with buffer solution (50 mM Tris-HCl, pH 7.4, RT, 10 min) and then incubated with [^64^Cu]Cu‑NODAGA-ATV:4D9 (0.1 to 0.5 MBq) in phosphate buffer (pH 7, 60 min, RT). To verify specificity, a 1000-fold excess of unlabeled ATV:4D9 antibody (2000 µg) was added to 2 MBq of [^64^Cu]Cu‑NODAGA-ATV:4D9 (1.3 µg), and each section was incubated with 0.1 MBq of the tracer. Sections were rinsed first with cold Tris-HCl + 5% ethanol (pH 7.4, 1 × 5 min, 4 °C), distilled water (RT, 5 sec.) and then dried for 1 h at RT. For *ex vivo* autoradiography, one brain hemisphere was isolated, fixed with polymer gel (Tissue-Tek® O.C.T. Compound), and frozen at -20 °C. The brain tissues were cut in sagittal sections of 20 µm thickness using a Leica CM 1860 cryostate. All brain sections were exposed to a phosphor imaging plate and placed in a dark cassette. After 24 to 30 h exposure, the plate was scanned by a CR-Reader (CR35 BIO, DÜRR MEDICAL). The Aida Image Analyzer v 450 software was used for image analysis. A manually drawn region of interest (ROI) was placed in the cerebellum as a pseudo-reference tissue. After background subtraction, intensity normalization of all sections was performed by calculation of brain-to-cerebellum (CBL) ratios. Human brain sections were deparaffinized (xylene, xylene:EtOH 1:1, 100% EtOH, 95% EtOH, 70% EtOH, 50% EtOH, dH_2_O, 3 min each), unmasked via antigen retrieval (Tris/EDTA pH 9.0, heat-induced in a pressure cooker, boiling for 3 min), blocked (5% BSA in PBS/Triton (0.25% v/v) for 1 h at RT), incubated with [^64^Cu]Cu‑NODAGA-14D3 (0.1 MBq or an added 1000-fold excess of unlabeled 14D3 antibody, 1 h at RT), and washed (PBS, 3×10 min). Subsequent steps were carried out in accordance with the methodology for murine brain sections. For human brain sections, white matter (WM) was used as the internal reference region, and cortex-to-white matter ratios are given.

### Small animal PET

Mice from cohorts 1 and 2 were administered 13.1 ± 3.2 MBq [^64^Cu]Cu‑NODAGA-4D9 (n = 29) or 10.2 ± 1.3 MBq [^64^Cu]Cu‑NODAGA-ATV:4D9 (n = 30) (corresponding to 8.1 ± 1.1 µg and 10.2 ± 1.9 µg per mouse) in 150 µL phosphate buffer by intravenous injection through the tail vein. Table 1 indicates which tracer was applied to which individual mouse model. The PET scan (Mediso Nanoscan PET/CT, 70 kvp/650 µA, exposure time 300 ms, Helical 1.0 pitch, with coincidence mode 1-5 in 1 scan position) was conducted 2 h, 20 h, and 40 h post-injection (n = 8 per time point, 30 min emission time). The mice were continuously anesthetized with isoflurane during the scan. PET images were reconstructed using the Tera Tomo^TM^ 3D algorithm (4 iterations and 6 subsets). The resulting images were analyzed using PMOD (version 3.5; PMOD Technologies Ltd.). The CT and PET images of each mouse were aligned and registered to the magnetic resonance imaging (MRI) mouse brain atlas. An average image was generated for each group at 2 h, 20 h and 40 h p.i. Volumes-of-interest (VOIs) were manually placed in the hippocampus and cortex to assess tracer uptake by calculating the percentage of injected dose per gram of tissue (%ID/g). Cohen's d was calculated to estimate effect sizes. PET-to-biodistribution correlation analysis was performed using whole-brain voxel-wise SPM analysis (Wellcome Department of Cognitive Neurology, London, UK) in MATLAB (v2011-R2016) 45. The correlation was considered significant if p < 0.05. Data-driven cluster VOIs from SPM analysis were used for regression coefficient calculation and comparison of tracer uptake (%ID/g) between all groups.

Mice from cohort 3 were administered 38.1 ± 1.2 MBq [^64^Cu]Cu‑NODAGA-ATV:4D9 (n = 4) (corresponding 30.5 ± 1.0 µg per mouse) into the tail vein. PET imaging was performed and analyzed at 20 h p.i. following the protocol described above. PET signal increases of cohort 3 (in contrast to WT;TfR^mu/hu^ of cohort 2B) were quantitatively compared with radioactivity of isolated microglial cells (single cell Radiotracing (scRadiotracing), see detailed description below) in all four individual App^SAA^;TfR^mu/hu^ mice. To this end, 7.4 × 10^6^ microglia built the basis for estimation of microglia cell numbers in aged App^SAA^;TfR^mu/hu^ mice.

### Biodistribution

After PET acquisition, a solution of ketamine 10% (m/v) and xylazine 2% (m/v) in 0.9% NaCl was administered intraperitoneally to each mouse. Mice were intracardially perfused using 40 mL PBS buffer. At 2 h and 20 h post-injection, the brain (either the whole brain or one hemisphere) was collected, and the tracer enrichment was measured using a gamma counter (n = 6). Additionally, a whole-body biodistribution study was carried out at 40 h post-injection (n = 6), including brain, liver, lungs, heart, blood, kidneys, muscle, pancreas, spleen and bone. The tracer enrichment in the individual organs was measured using a gamma counter (HIDEX AMG Gamma Counter, version 1.6.0.0, counting window from 450 to 570 keV; 1 min; including mass measurement) and expressed as %ID/g.

### Single cell Radiotracing

App^SAA^;TfR^mu/hu^ mice were injected with 38.1 ± 1.2 MBq [^64^Cu]Cu‑NODAGA-ATV:4D9 (corresponding 30.5 ± 1.0 µg per mouse) into the tail vein. At 20 h p.i., the mice were euthanized via cervical dislocation and the mouse brains were removed and briefly washed and stored in cold Dulbrecco's phosphate-buffered saline (D-PBS). The brains were placed in a 10 mm petri dish containing 2 mL cold D-PBS under a dissecting microscope (Leica Microsystems M80, Wetzlar, Germany), and meninges were gently detached from the skull and removed using fine forceps (Dumont, 11254-20). Immunomagnetic cell separation (MACS) was performed as described previously 46, 47 with slight modifications. Adult Brain Dissociation Kit, mouse and rat (Miltenyi Biotec, 130-107-677) was used for brain tissue dissociation according to the supplier's instructions. The brains were cut into small pieces and dissociated with enzyme mix 1 and 2 using gentleMACS Octo Dissociator with heaters (Miltenyi Biotec, 130-096-427). The cell suspension was applied to a preconditioned 70 μm MACS SmartStrainer (Miltenyi Biotec, 130-110-916) and debris was removed by using Debris Removal Solution. Microglia were labeled using CD11b MicroBeads (Miltenyi Biotec, 130-049-601) and subsequently separated using two-column sorting on the autoMACS Pro Separator (Miltenyi Biotec, 130-092-545). CD11b-enriched and depleted fractions were collected, centrifuged (4 °C, 400 × g, 10 min) and the supernatant was aspirated. The cell pellets were resuspended in 100 µL cold D-PBS and incubated with 2 µL anti-mouse CD11b antibody, (Miltenyi Biotec, 130-113-238) at 4 °C for 10 minutes in the dark. Cells were washed with 1 mL D-PBS, centrifuged (4 °C, 300 × g, 10 min), and the supernatant was aspirated. The cell pellets of the CD11b-enriched fractions were resuspended in 500 µL D-PBS and the depleted fractions were resuspended in 1 mL D-PBS. Radioactivity of the samples was measured in a gamma counter (Hidex AMG Automatic Gamma Counter, Mainz, Germany) and decay corrected to 20 h post tracer injection. Absolute cell counts and purity of CD11b-enriched cell pellets in each sample were acquired on a MACSQuant10 analyzer (Miltenyi Biotec, 130-105-100).

### Immunohistochemistry of human brain sections

Human brain tissue for *in vitro* autoradiography and images of stained human brain sections were provided by the Center of Neuropathology and Prion Research, LMU Munich, Germany. Approval for *ex vivo* tissue staining was obtained from the local ethics committee (application number 19-244).

AD brain tissues were taken from six patients with following characteristics: ABC score A3/B3/C3 (which is an amyloid β score modified from Thal of phases 4 or 5, neurofibrillary tangles stage modified from Braak of stage V or VI, and neuritic plaque score modified from CERAD of score frequent), 63-88 years old, n = 4 female and n = 2 male. The TREM2 polymorphisms were unknown for all cases.

Immunohistochemistry, including counterstaining with hematoxylin, was performed on 5 µm thick paraffin sections using a Ventana BenchMark ULTRA (Roche) according to the instructions of the manufacturer. Primary antibody was mouse anti-TREM2 (diluted 1:200; Merck MABN1534). Pretreatment for antibody was boiling in CC2 buffer (Roche) for 48 min. The diaminobenzidine/peroxidase based detection system was optiView (Roche). Immunohistochemical stains were scanned with a Zeiss Axio Scan.Z1 using a 20x objective. Zeiss blue and ImageJ were used for quantification. TREM2 was quantified as the % area.

### Statistical analysis

GraphPad Prism (version 9) was used to carry out the statistical analysis. Biodistribution and PET (frontal cortex and hippocampus and data-driven regions) were analyzed using one-way ANOVA and Tukey's post hoc test to adjust for multiple comparisons. Ratios of autoradiography experiments (*in vitro* and *ex vivo*) were compared between genotypes using an unpaired t-test. A regression analysis between biodistribution and PET was performed using statistical parametric mapping (SPM) using SPM12 routines (Wellcome Department of Cognitive Neurology, London, UK) implemented in MATLAB (version 2016). Individual %ID images were subject to a linear regression analysis with biodistribution tracer uptake as a vector in the pooled cohort of all genotypes. Data-driven clusters were selected at a threshold of p < 0.005 uncorrected, k > 20 voxels. Pearson's coefficient of determination (R^2^) was calculated for VOI-based PET to biodistribution correlation analysis. Similarity of quantitative brain radiotracer uptake and microglial radiotracer uptake was assessed with a paired t-test.

## Results

### ^64^Cu-labeling of 4D9 and ATV:4D9 antibodies yields radiotracers of high stability

Antibodies were modified via covalent attachment of the *p*-NCS-benzyl-NODAGA chelator to lysine residues on the surface of the antibody. 1-2 chelators were bound to the antibodies as determined with an arsenazo assay (Figure S1) 44. This spectrophotometric assay allows for the determination of the chelator-antibody ratio. The number of chelators per antibody should be kept on a low level to avoid alterations of biodistribution and impairment of the antibody's immunoreactivity. High radiochemical purity (RCP), radiochemical yield (RCY) and specific activity (A_s_) were obtained for both [^64^Cu]Cu‑NODAGA-4D9 and [^64^Cu]Cu‑NODAGA-ATV:4D9 (Table 2). The RCP was determined using radio-TLC, radio-HPLC and SDS-PAGE (Figure S2). The results confirmed that there was no chelator-bound or free [^64^Cu]CuCl_2_ present in the injectable solutions.

The stability of [^64^Cu]Cu‑NODAGA-ATV:4D9 was assessed by incubation in murine plasma for 48 h at 37 °C. Samples were taken and analyzed by SEC-HPLC showing that the tracer remained stable over 48 h. We performed *ex vivo* stability analysis using SDS-PAGE and radio-TLC of plasma samples from [^64^Cu]Cu-NODAGA-ATV:4D9 and [^64^Cu]Cu-NODAGA-4D9 injected WT mice 20 h p.i. SDS-PAGE revealed intact radiotracer and the absence of any other proteins that might have been radiolabeled via trans-chelation whereas radio-TLC confirmed the absence of unbound copper-64 (Figure S3).

### Antibody-based TREM2 tracer exhibits full functionality and high specificity

**Figure 1:**
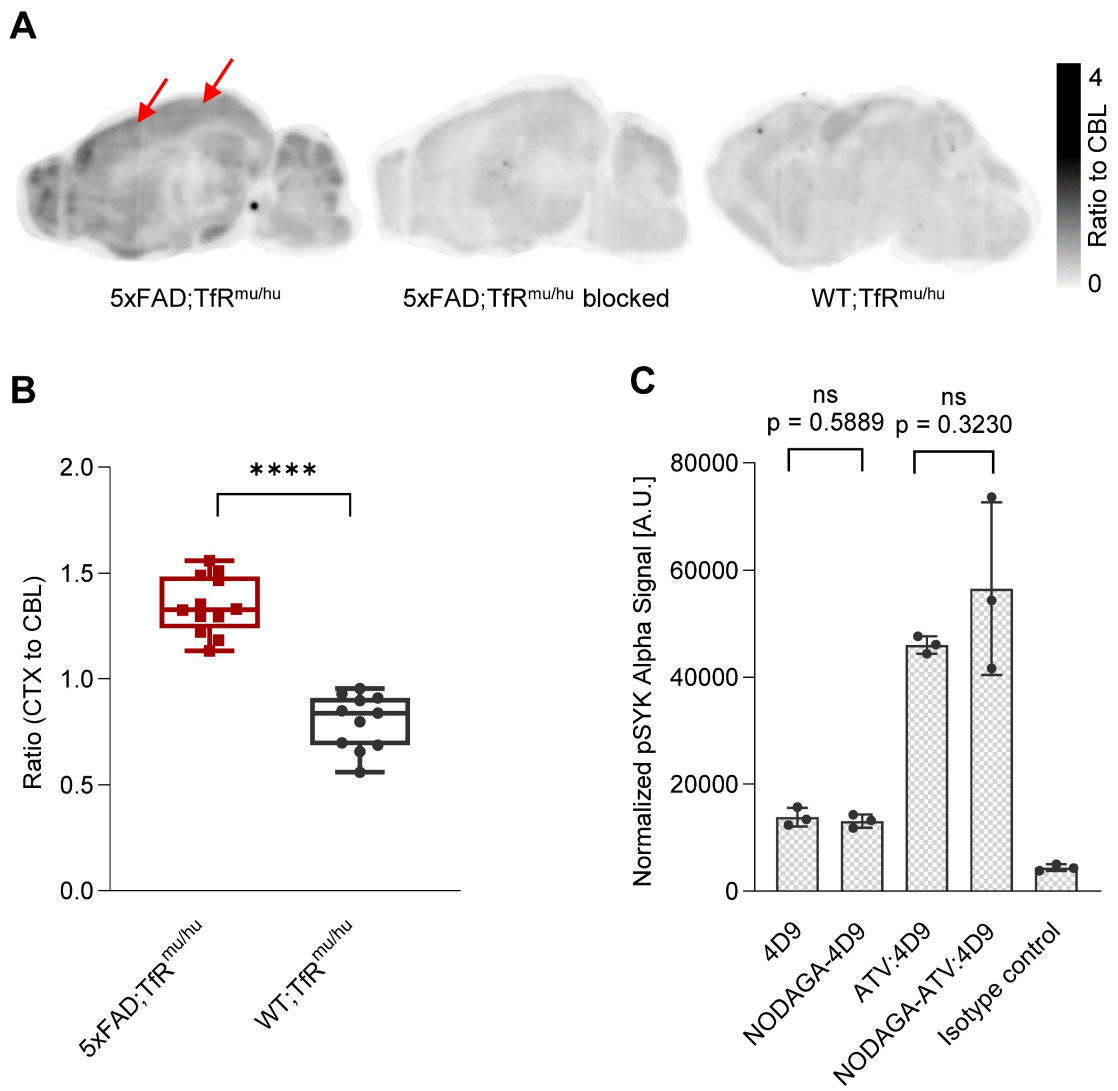
***In vitro* autoradiography experiments.** (**A**) Sagittal sections show autoradiography results from 5xFAD;TfR^mu/hu^ brain, upon blocking with 1000-fold excess cold ATV:4D9 antibody, and WT;TfR^mu/hu^ brain. Arrows indicate increased uptake of [^64^Cu]Cu‑NODAGA-ATV:4D9 in the frontal cortex. (**B**) The brain sections of 5xFAD;TfR^mu/hu^ (4 mice, 12 sections) revealed higher cortex-to-cerebellum ratio compared to WT;TfR^mu/hu^ (3 mice, 11 sections) mice. Unpaired t-test, p < 0.0001 (****), boxplot min to max. (**C**) Quantification of pSYK levels by AlphaLISA (normalized to protein concentration) in lysates from HEK293 Flp-In cells that stably overexpress mouse TREM2 and mouse DAP12. Cells were stimulated with 4D9, NODAGA-4D9, ATV:4D9, NODAGA-ATV:4D9, and an isotype control. The experiment was performed once including n = 3 technical replicates. Unpaired t-test, (n = 3, mean ± SD).

Brain sections (4 mice, 12 sections) from 5xFAD;TfR^mu/hu^ mice were used for *in vitro* autoradiography using [^64^Cu]Cu‑NODAGA-ATV:4D9 (Figure 1A). The images revealed high tracer enrichment in the frontal cortex and the hippocampus, where strong microglial activation occurs in this mouse model 48-51. When brain sections from 5xFAD;TfR^mu/hu^ (5 mice, 20 sections) were treated with [^64^Cu]Cu‑NODAGA-ATV:4D9 in the presence of 1000-fold excess of cold ATV:4D9 antibody, tracer accumulation was significantly reduced, indicating that the tracer binds specifically to its target (Figure 1A). Compared to 5xFAD;TfR^mu/hu^ mice, the brain sections (3 mice, 11 sections) of WT;TfR^mu/hu^ mice revealed less pronounced tracer enrichment in cortical and hippocampal areas and a rather equally distributed accumulation pattern (Figure 1A). Quantitative readout from *in vitro* experiments revealed a cortex-to-cerebellum ratio of 1.35 ± 0.14 (mean ± SD) for 5xFAD;TfR^mu/hu^ and 0.80 ± 0.13 for WT;TfR^mu/hu^ mice (Figure 1B).

To investigate if the antibody modification affects TREM2 binding, NODAGA-modified antibodies were compared with the native antibodies using a p-SYK signaling assay (Figure 1C). Antibody-mediated activation of p-SYK signaling downstream of TREM2 was not reduced, indicating that TREM2 binding is not impaired. Higher potency of ATV:4D9 compared to 4D9 antibodies can be explained by the concomitant expression of hTfR on TREM2-DAP12 overexpressing HEK293 cells enabling increased cross-linking of TREM2 and enhanced endosomal signaling 41.

### hTfR-mediated transcytosis facilitates higher brain uptake of ^64^Cu-labeled TREM2 antibodies in mice

**Figure 2:**
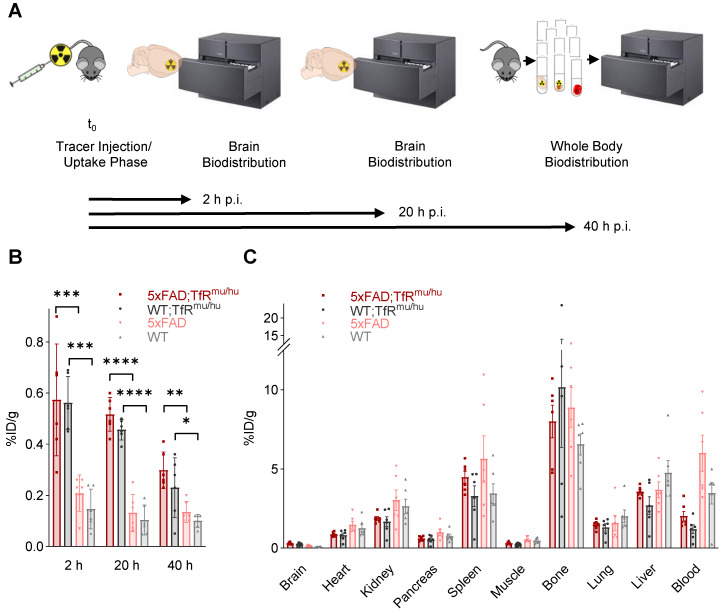
** Biodistribution experiments.** (**A**) Schematic representation of the biodistribution workflow. (**B**) Decay-corrected brain uptake of [^64^Cu]Cu‑NODAGA-ATV:4D9 in 5xFAD;TfR^mu/hu^ and WT;TfR^mu/hu^, as well as [^64^Cu]Cu‑NODAGA-4D9 in 5xFAD and WT mice, was determined after intracardial perfusion at 2 h, 20 h, and 40 h p.i. One-way ANOVA/Tukey's multiple comparison test, p ≤ 0.05 (*), p ≤ 0.01 (**), p ≤ 0.001 (***), and p ≤ 0.0001 (****), mean ± SD. Non-decay-corrected data are presented in Figure S5. (**C**) Whole-body biodistribution at 40 h p.i.

Brain uptake of [^64^Cu]Cu‑NODAGA-ATV:4D9 and [^64^Cu]Cu‑NODAGA-4D9 was measured after intracardial perfusion using gamma counting at 2 h, 20 h and 40 h p.i. (n = 6) (Figure 2A). Brain uptake of [^64^Cu]Cu‑NODAGA-ATV:4D9 in 6-7 months old 5xFAD;TfR^mu/hu^ mice was up to 4.6-fold higher compared to [^64^Cu]Cu‑NODAGA-4D9 in age-matched 5xFAD mice (Figure 2B, Table S1) and remained at a high level over 20 h. Similar results were obtained when comparing WT;TfR^mu/hu^ and WT mice at the same age. To break down the relative proportion of hTfR binding to total binding, we determined brain uptake of [^64^Cu]Cu-NODAGA-ATV:ISO (isotype, reflecting hTfR binding only) and [^64^Cu]Cu-NODAGA-ATV:4D9 (reflecting hTfR and TREM2 binding) in WT;TfR^mu/hu^ mice 20 h p.i. via biodistribution (Figure S4). We found that 80% of the signal in the brain is attributed to hTfR binding.

Whole-body biodistribution (Figure 2C, Table S2) of perfused mice was performed at 40 h p.i. (n = 6). High tracer uptake was observed in bone, spleen, liver and kidney. Uptake levels were low in heart, pancreas and lung, and lowest in muscle. With the exception of the brain, 5xFAD;TfR^mu/hu^ mice tended to exhibit lower tracer uptake in all organs compared to 5xFAD mice. The same trend could be observed in WT;TfR^mu/hu^ mice compared to WT mice (except bone). Blood retention assessed before perfusion was considerably higher in mice without hTfR expression compared to 5xFAD;TfR^mu/hu^ and WT;TfR^mu/hu^ mice. In summary, biodistribution data confirm the hypothesis that organ biodistribution was mainly driven by TfR binding.

### TREM2 PET shows elevated signals in mice with increased TREM2 levels

**Figure 3:**
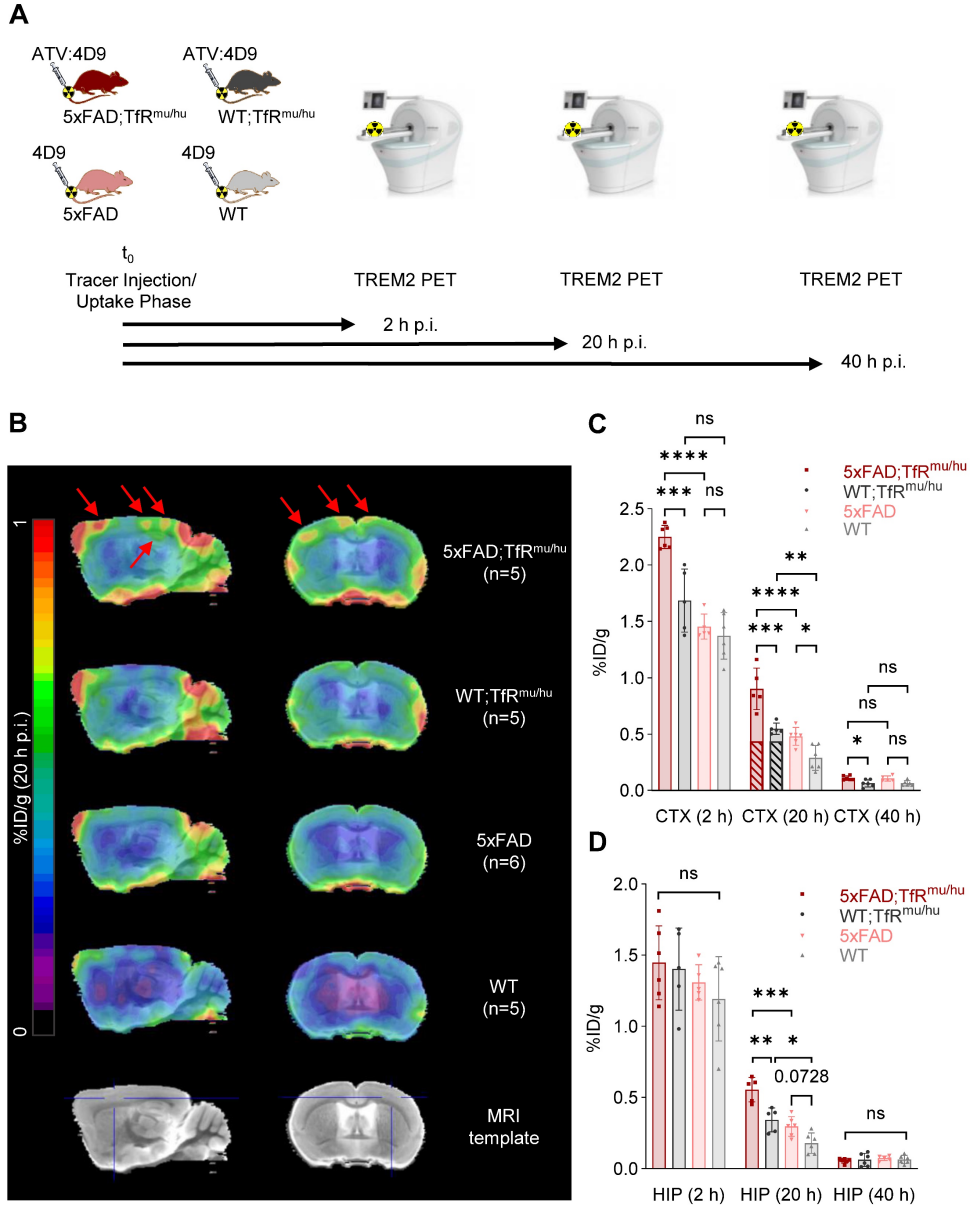
** TREM2 PET imaging in 5xFAD and wild-type mice.** (**A**) Schematic representation of the PET/CT workflow. (**B**) Group average PET images of 5xFAD;TfR^mu/hu^, 5xFAD, WT;TfR^mu/hu^ and WT mouse brains at 20 h p.i. overlaid on an MRI template. Red arrows indicate highest uptake in the frontal cortex and the hippocampus of 5xFAD;TfR^mu/hu^ animals. (**C, D**) Quantitative tracer uptake (%ID/g) in predefined VOIs of the frontal cortex (CTX) and the hippocampus (HIP). Striped bars illustrate the fraction of hTfR-related binding determined by biodistribution using ^64^Cu-labeled ATV:ISO (Figure S4). One-way ANOVA/Tukey's multiple comparison test, p ≤ 0.05 (*), p ≤ 0.01 (**), p ≤ 0.001 (***), and p ≤ 0.0001 (****), mean ± SD.

Next, we investigated if ^64^Cu-labeled 4D9 and ATV:4D9 antibodies are suitable radiotracers to visualize TREM2 *in vivo* using small animal PET imaging at 2 h, 20 h and 40 h p.i. (Figure 3A). Figure 3B presents group average PET images generated 20 h p.i. (images at 2 h p.i. and 40 h p.i. are shown in Figure S6). Regions of interest (VOIs) were predefined in the frontal cortex and the hippocampus based on the Mirrione atlas 52, 53 with adjustment to exclude spill-in signals from skull, which was characterized by high biodistribution. A significantly higher uptake was found in the frontal cortex (Figure 3C) and in the hippocampus (Figure 3D) at 20 h p.i. of 5xFAD;TfR^mu/hu^ compared to WT;TfR^mu/hu^ mice at 6-7 months of age (Tables S3 and S4). Considering the fraction of hTfR-related binding determined by biodistribution, which is comparable in both animal models 41, it is evident that the tracer is able to unveil TREM2-dependent signal enhancement. Elevated TREM2 PET signals were consistent with higher TREM2 protein levels found in 5xFAD brain lysates compared to wild-type controls (Figure S7). No difference in tracer uptake was observed at the 40 h p.i. time point, likely due to a decay-related decrease in signal-to-noise ratios. In addition, effect sizes expressed as Cohen's d were high comparing 5xFAD;TfR^mu/hu^ vs WT;TfR^mu/hu^ and 5xFAD;TfR^mu/hu^ vs 5xFAD mice at 2 h and 20 h p.i. in cortex and 20 h p.i. in the hippocampus (Table S5). A comparison of ^64^Cu-labeled ATV:4D9 to a compilation of PET data from multiple TSPO tracers revealed that TREM2 PET resulted in higher %ID/g and SUVR ratios than TSPO PET (Figure S8, Tables S6 and S7).

**Figure 4:**
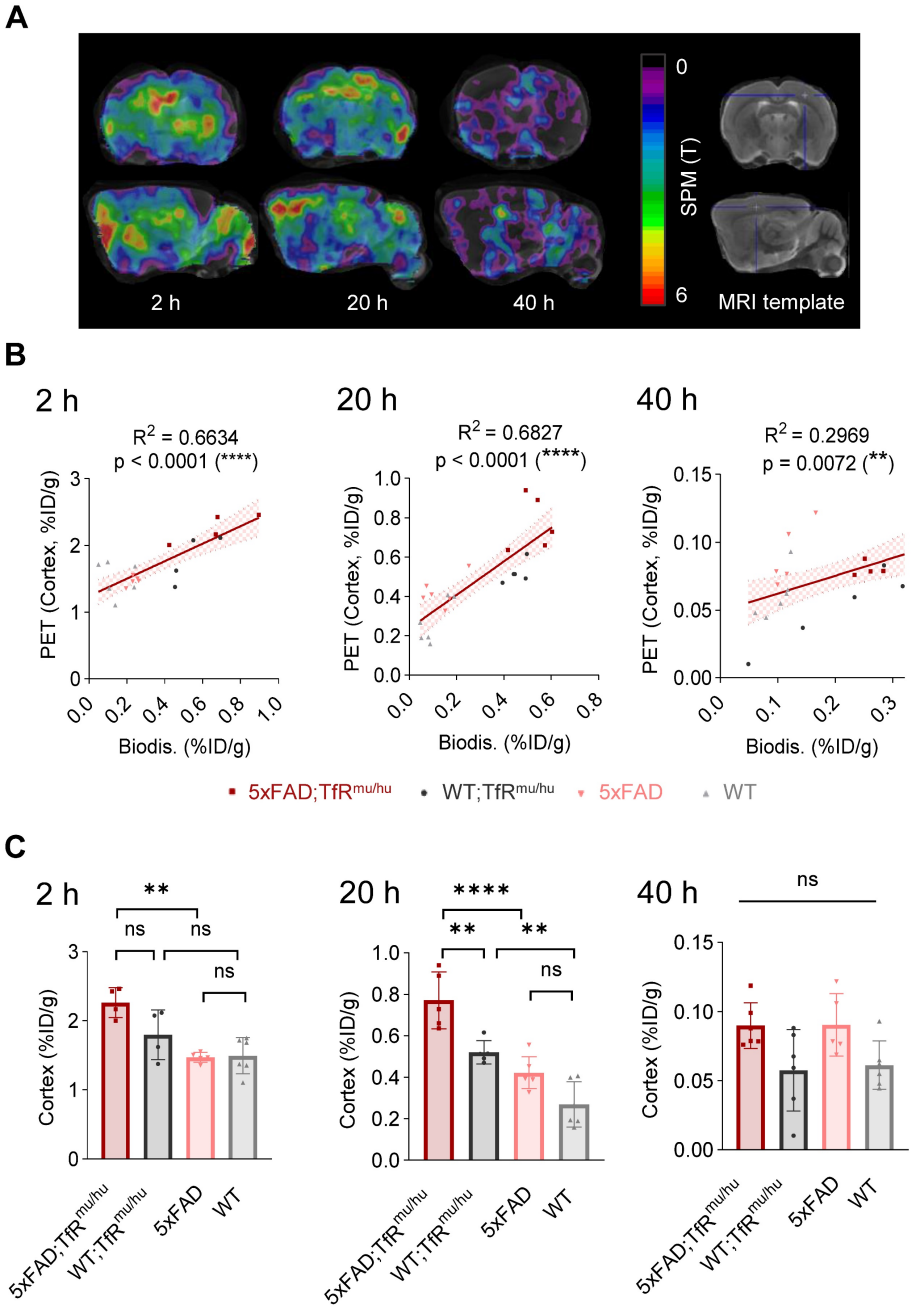
** PET-to-biodistribution associations.** (**A**) Images from voxel-wise regression analysis of biodistribution brain uptake with PET images using statistical parametric mapping (SPM). Color scale shows TREM2 PET voxels with highest correlation to biodistribution in red using all four genotypes. (**B**) TREM2 PET signals in data-driven cortical cluster VOIs derived from the regression analysis in correlation with brain uptake from biodistribution at 2 h, 20 h and 40 h p.i. (linear regression, α = 0.05, 95% CI), (**C**) Group comparison of TREM2 PET results in data-driven cortical cluster VOIs across genotypes. One-way ANOVA/Tukey's multiple comparison test, p ≤ 0.01 (**), p ≤ 0.001 (***), and p ≤ 0.0001 (****), mean ± SD.

To address spill-in from skull into cortex due to limited spatial resolution of small animal PET, we performed data-driven identification of cortical brain regions where PET signals are derived from TREM2 target engagement. To this end, we performed a voxel-wise correlation analysis between brain biodistribution and brain uptake in TREM2 PET. Statistical maps derived from regression analyses revealed cluster VOIs, which were most strongly associated with uptake values from biodistribution. Accordingly, elevated radiotracer uptake in whole brain of 5xFAD;TfR^mu/hu^ mice was driven by voxels in the frontal cortex (Figure 4A), which showed an excellent linear correlation with biodistribution, pronounced at 20 h p.i. (Figure 4B and Table S8). In this optimized target region, 5xFAD;TfR^mu/hu^ mice revealed significantly higher uptake than the WT;TfR^mu/hu^ controls at 20 h p.i. (Figure 4C and Table S9). At 2 h p.i., the correlation between biodistribution and PET was also high, but only a trend towards higher uptake in 5xFAD;TfR^mu/hu^ compared to WT;TfR^mu/hu^ mice was detected. The correlation between biodistribution and PET was lower at 40 h p.i. and no statistically significant differences were obtained. Altogether, these data supported 20 h p.i. as the most robust time point for TREM2 PET imaging with antibody-labeled tracers.

### *Ex vivo* autoradiography confirms the regional TREM2 PET distribution pattern of tracer uptake in the brain

**Figure 5:**
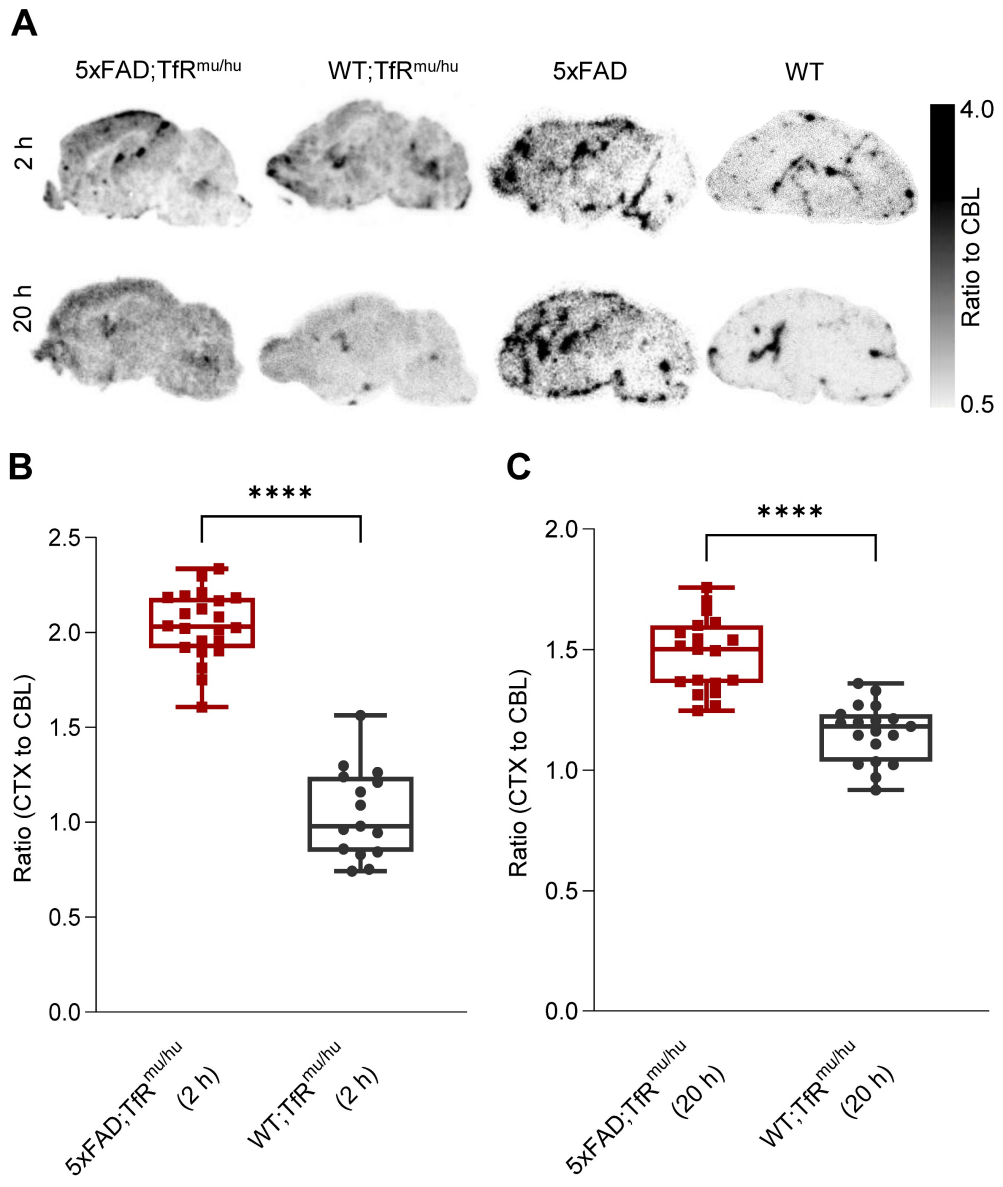
***Ex vivo* autoradiography confirmation of regional TREM2 PET signals.** (**A**) *Ex vivo* autoradiography of 5xFAD;TfR^mu/hu^, WT;TfR^mu/hu^, 5xFAD and WT brain sections at 2 h and 20 h p.i. (**B,C**) Higher cortex-to-cerebellum ratios were observed in 5xFAD;TfR^mu/hu^ mice compared to WT;TfR^mu/hu^ mice at the 2 h p.i. time point (5xFAD;TfR^mu/hu^: 2 mice, 22 sections; WT;TfR^mu/hu^: 2 mice, 15 sections; unpaired t-test, p < 0.0001 (****), boxplot min to max) and the 20 h p.i. time point (5xFAD;TfR^mu/hu^: 2 mice, 19 sections; WT;TfR^mu/hu^: 2 mice, 19 sections; unpaired t-test, p < 0.0001 (****), boxplot min to max).

*Ex vivo* autoradiography was performed from brain sections of perfused 5xFAD;TfR^mu/hu^, WT;TfR^mu/hu^, 5xFAD and WT mice at 2 h and 20 h p.i. as a validation experiment for *in vivo* patterns as obtained by TREM2 PET imaging (Figure 5A). The cortex-to-cerebellum ratio was calculated as a quantitative readout. Obtained CTX/CBL values were 2.04 ± 0.18 (mean ± SD) for 5xFAD;TfR^mu/hu^, 1.05 ± 0.24 for WT;TfR^mu/hu^ mice at 2 h p.i. (p < 0.0001) (Figure 5B), and 1.48 ± 0.15 for 5xFAD;TfR^mu/hu^ and 1.16 ± 0.12 for WT;TfR^mu/hu^ mice at 20 h p.i. (p < 0.0001) (Figure 5C). Patterns of tracer retention in autoradiography mirrored data-driven clusters of highest TREM2 tracer binding in PET.

### scRadiotracing confirms high cellular selectivity of [^64^Cu]Cu-NODAGA-ATV:4D9 for microglia and validates TREM2 PET in a second mouse model of amyloidogenesis

**Figure 6:**
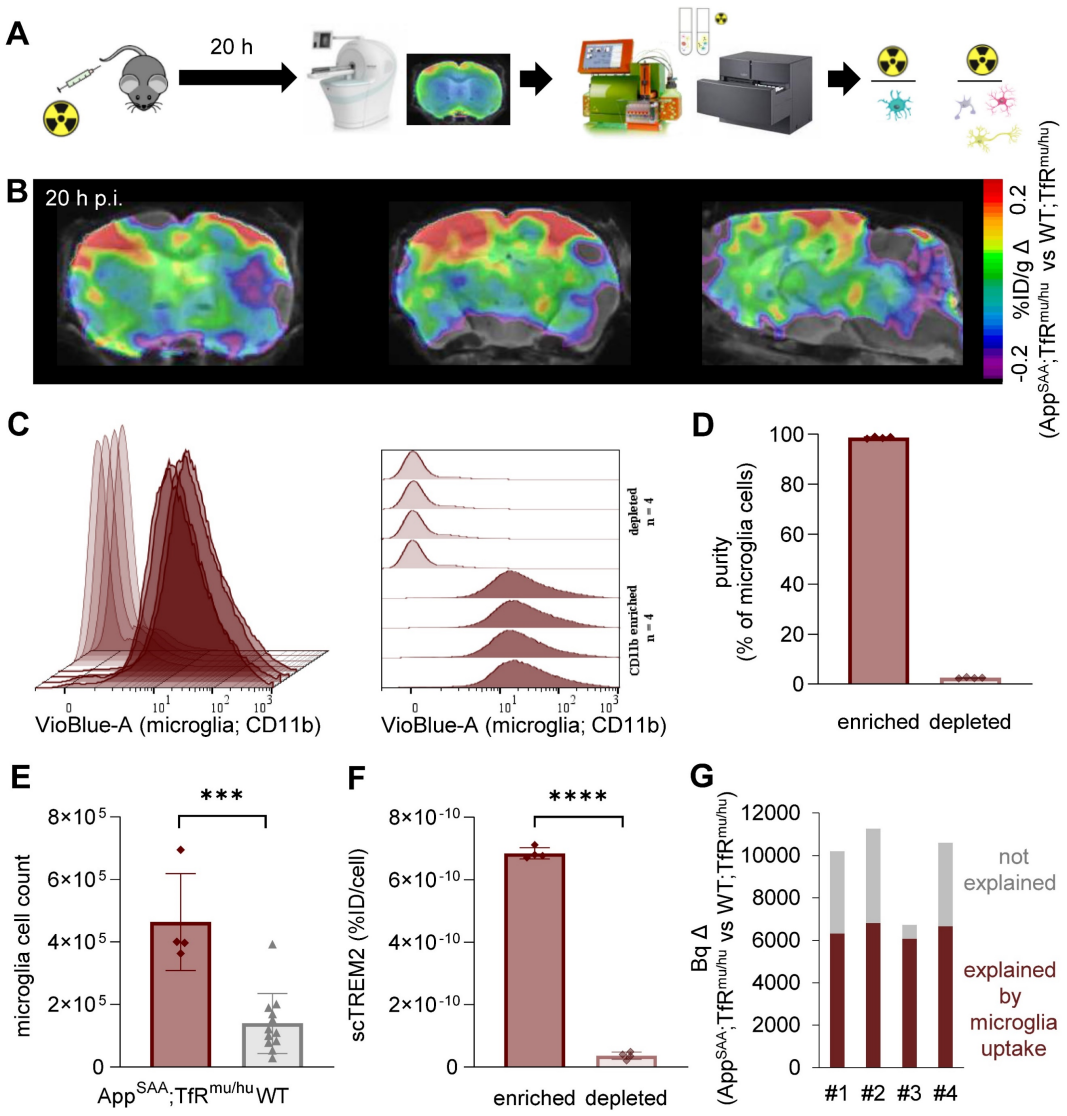
** scRadiotracing demonstrates specificity of [^64^Cu]Cu-NODAGA-ATV:4D9 to microglia.** (**A**) Experimental workflow, including TREM2 PET at 20 h p.i., brain dissociation and cell sorting as well as flow cytometry and gamma emission recording to calculate radioactivity per cell (microglia = turquoise, astrocyte = pink, neuron = yellow, oligodendrocyte = gray). (**B**) TREM2 PET results are shown as axial (frontal cortex, hippocampus) and sagittal (forebrain, hindbrain) regional difference maps (n = 4 App^SAA^;TfR^mu/hu^ mice compared to n = 5 WT;TfR^mu/hu^ of Fig.3) projected upon an MRI template. (**C, D**) Flow cytometry indicates high purity of CD11b-positive cells in microglia-enriched fractions and absence of CD11b-positive cells in the microglia-depleted fractions of n = 4 individual App^SAA^;TfR^mu/hu^ mice. Data are shown as mean fluorescence intensity. (**E**) Relative cellular abundance of microglial cells in App^SAA^;TfR^mu/hu^ (n = 4) mice compared to WT (n = 12) mice. Unpaired t-test, p = 0.0002 (***), mean ± SD. (**F**) TREM2 radiotracer uptake of microglia-enriched vs microglia-depleted (i.e. mixed fraction of neurons, astrocytes, oligodendrocytes) fractions in App^SAA^;TfR^mu/hu^ mice, confirming high specificity to microglia. Unpaired t-test, p < 0.0001 (****), mean ± SD. (**G**) Estimation of the PET signal percentage which is explained by microglial uptake as a product of microglial abundance and microglial tracer uptake per cell. Based on (**E**) and 7.4 × 10^6^ microglia in WT brains taken from literature, we calculated 24.7 × 10^6^ microglia in App^SAA^;TfR^mu/hu^ brains.

To validate the cellular origin (i.e. microglial specificity) of TREM2 PET signals in the brain, we applied a novel technique of cell sorting after PET tracer injection and PET imaging 47 in an independent mouse model of amyloidogenesis, i.e. App^SAA^;TfR^mu/hu^ mice (Figure 6A and Figure S9) 54. With this approach, we detected [^64^Cu]Cu‑NODAGA-ATV:4D9 in microglial and non-microglial cells of the brain at the time of PET imaging. This mouse model has a humanized apical domain of TfR where the chimeric receptor is referred to as TfR^mu/hu^, in addition to the presence of β-amyloid plaques with concomitant increase in TREM2 levels 54. TREM2 PET revealed an enhanced signal predominantly in frontal cortex and hippocampal brain regions of aged App^SAA^;TfR^mu/hu^ mice (Figure 6B, group-average PET images are shown in Figure S10). Immunomagnetic cell sorting allowed isolation of a microglia-enriched fraction with 98% CD11b^+^ and microglia-depleted cell pellets with nearly complete absence of CD11b^+^ cells (Figure 6C-D). App^SAA^;TfR^mu/hu^ mice showed a 3.3-fold increase in microglia in whole brain (Figure 6E). Gamma emission recordings indicated a strong signal in the microglia-enriched fraction but negligible signal in the microglia-depleted fraction, confirming the cellular specificity of [^64^Cu]Cu‑NODAGA-ATV:4D9 binding to microglia (Figure 6F). Furthermore, we estimated the microglia-related increase in brain radiotracer uptake in PET of App^SAA^;TfR^mu/hu^ vs. WT;TfR^mu/hu^ controls. To do so, microglial cell number was multiplied by the activity per microglial cell from scRadiotracing experiments to obtain the signal derived from microglial uptake in App^SAA^;TfR^mu/hu^ mice (dark red bars in Figure 6G), which explains 67% of the PET signal increase in App^SAA^;TfR^mu/hu^ mice compared to WT;TfR^mu/hu^ controls (Figure 6G).

### Labeled anti-human TREM2 antibody indicates potential for translation to clinical TREM2 PET imaging

**Figure 7:**
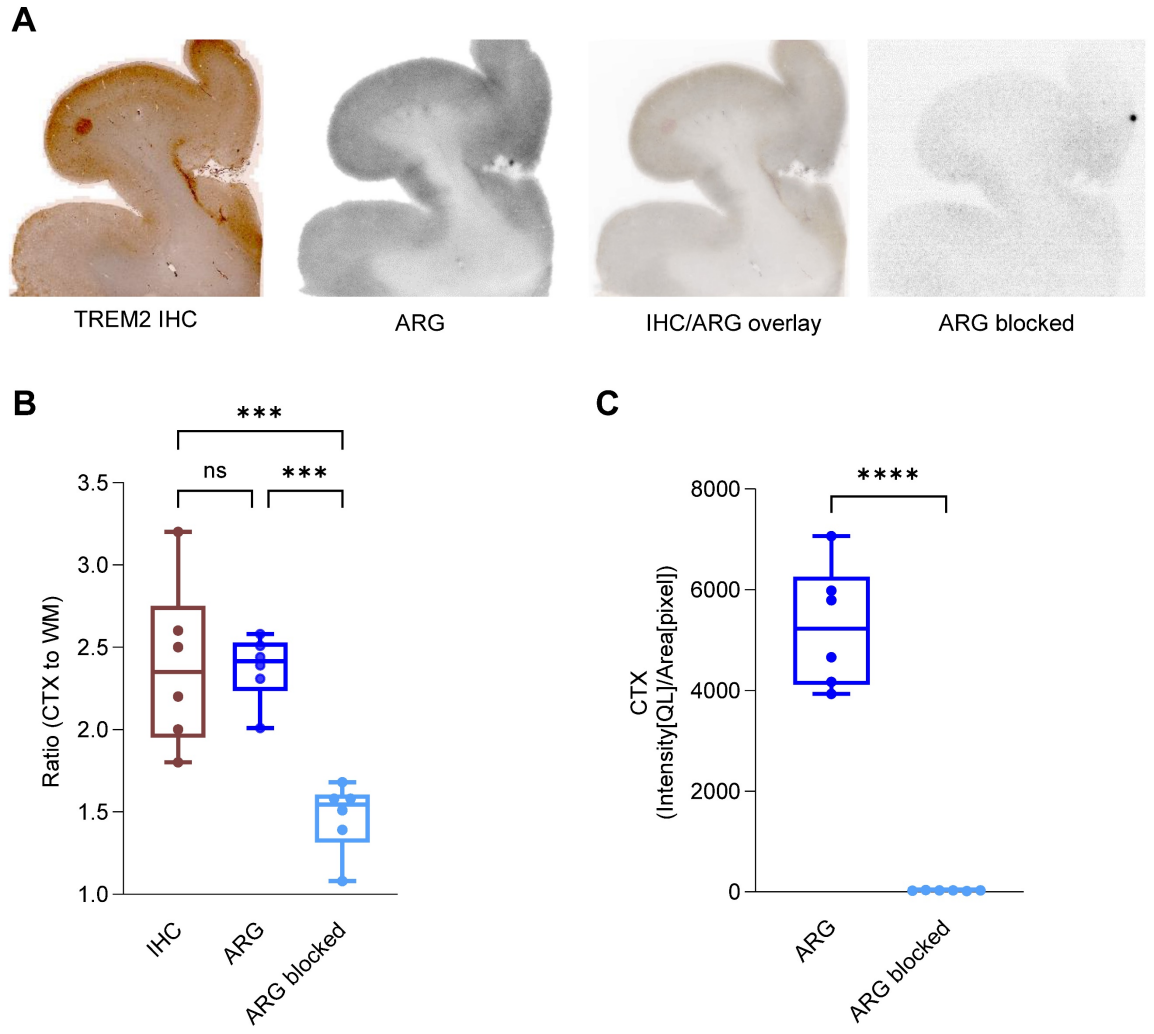
** [^64^Cu]Cu‑NODAGA-14D3 ARG signal represents TREM2 IHC signal in Alzheimer's disease patients.** (**A**) Representative TREM2 immunohistochemistry (IHC) and *in vitro* autoradiography (ARG) of frontal brain sections derived from a patient with Alzheimer's disease (AD) revealed cortical binding of [^64^Cu]Cu‑NODAGA-14D3. TREM2 IHC and tracer binding in ARG co-localized. Autoradiography of a blocked brain slice demonstrated a negligible signal. (**B**) Cortex-to-white matter ratios were consistent in IHC and ARG and significantly higher than in blocked ARG (One-way ANOVA/Tukey's multiple comparison test, p ≤ 0.001 (***), boxplot min to max). (**C**) ARG-blocking resulted in a cortical signal reduction (unpaired t-test, p < 0.0001 (****), boxplot min to max).

We next investigated the translation of our promising preclinical findings into clinical applications. We conducted immunohistochemistry and *in vitro* autoradiography on human brain sections of patients with AD. The 14D3 antibody 55 targeting human TREM2 (Figure S11) was analogously modified with the chelator NODAGA and labeled with copper-64 (RCP > 99%, measured by radio-TLC, n = 2) in accordance with the techniques and methods established for 4D9. 14D3 exclusively binds full-length TREM2 at the cleavage site of human TREM2, in contrast to 4D9, which also binds sTREM2. Further, this antibody was not ATV-enabled, hence does not contain a binding site for the human TfR. TREM2 immunohistochemistry and [^64^Cu]Cu‑NODAGA-14D3 autoradiography co-localized in adjacent sections, with enriched binding in the cortex but not in the subcortical white matter of the brain sections (Figure 7A), suggesting high regional TREM2 expression associated with AD pathology, including Aβ plaque and Tau protein (Figure S12). No significant differences between cortex-to-white matter ratios in immunohistochemistry (2.35 ± 0.46, median ± SD) and autoradiography (2.42 ± 0.18, median ± SD) confirmed the consistency of both methods to detect TREM2 (Figure 7B). Autoradiography of blocked brain sections with a 1000-fold excess of the 14D3 antibody demonstrated an almost negligible signal (Figure 7C) and only a minimal unspecific binding (Figure 7A). These preliminary findings suggest that the ATV-enabled 14D3 antibody might be a potential candidate that can be tested in models for subsequent translation of TREM2 PET imaging into the clinic.

## Discussion

TREM2 has emerged as a highly relevant molecular biomarker and therapeutic target in various indications 56, since it is associated with critical functions of macrophages and microglia in disease. We present a proof of concept for a novel imaging tool to detect TREM2-expressing microglia by direct target engagement of TREM2 antibodies. We developed two radiotracers using ATV:4D9 and 4D9 antibodies targeting mouse TREM2 12, 41. We showed that the potency of both tracers remained unchanged after chemical modification, and [^64^Cu]Cu-NODAGA-ATV:4D9 was stable in murine plasma for 48 hours. The specificity of [^64^Cu]Cu-NODAGA-ATV:4D9 was proven in a blocking experiment in the presence of an excess amount of unlabeled antibody. *In vivo* TREM2 PET revealed significantly elevated TREM2 binding in a mouse model of amyloid pathology compared to wild-type mice, which was validated by *ex vivo* autoradiography. Moreover, the cellular specificity of [^64^Cu]Cu-NODAGA-ATV:4D9 binding to microglia was demonstrated by cell sorting after *in vivo* radiotracer injection. Preliminary *in vitro* testing of an analogously copper-64 labeled human TREM2 antibody revealed promising binding patterns on AD post-mortem tissue.

*In vitro* autoradiography with brain sections of 5xFAD;TfR^mu/hu^ mice at 6-7 months of age showed elevated [^64^Cu]Cu‑NODAGA-ATV:4D9 uptake in frontal cortex and hippocampus compared to brain sections of WT;TfR^mu/hu^ mice. Amyloid pathology in 5xFAD mice manifests as early as 2 months of age and results in a high plaque load in cortical and hippocampal brain regions, which is accompanied by strong microglial activation 48, 49, 51. Therefore, high TREM2 levels are present in these brain regions, which is in line with our autoradiography results. When testing the developed radiotracers *in vivo*, [^64^Cu]Cu-NODAGA-ATV:4D9 revealed a 4-fold higher brain uptake in 5xFAD;TfR^mu/hu^ than [^64^Cu]Cu-NODAGA-4D9 in 5xFAD mice and 4.6-fold higher brain uptake in WT;TfR^mu/hu^ than [^64^Cu]Cu-NODAGA-4D9 in WT mice at 20 h p.i. due to the BBB penetrance of the ATV technology 41, which improved tracer delivery into the brain 41, 42. A literature review shows that the radiotracer concentration in the brain in our study is higher than what has been reported for bispecific constructs using TREM2 or Aβ targeting antibodies and scFv8D3 for TfR binding 8, 57. Even higher brain concentrations were achieved with smaller TfR-targeting bispecific tribody constructs with more than 1 %ID/g, which is comparable to small molecule tracers 58.

Transferrin receptor 1 (TfR1) expression is not limited to brain endothelial cells, where it plays a vital role in the uptake of transferrin-bound iron into cells across the BBB, but TfR1 is also expressed in bone marrow 41. Notably, peripheral biodistribution studies revealed high tracer uptake in bone. While TREM2 is expressed in microglia in the CNS, it is also present on peripheral myeloid cells. Consequently, TfR and TREM2 binding may be the cause of high tracer accumulation in the bone marrow. Accordingly, tissue retention of the copper-64 labeled 4D9 antibody in mice without hTfR apical domain knock-in was higher in most organs and blood than copper-64 labeled ATV:4D9 in mice with hTfR knock-in. We conclude that a considerable amount of ATV:4D9 localizes in bone due to TfR binding compared to 4D9 antibody, reducing blood retention and bioavailability of residual ATV:4D9 antibody to other organs. A tracer application requires only a minimal quantity of antibody, and thus, further investigations are needed to fully understand the impact of TfR binding for ATV:4D9 at these low doses.

Next, we evaluated if both tracers are suitable for TREM2 PET imaging with a focus on different time points and target region optimization. We were able to verify higher PET-derived tracer uptake in the frontal cortex and the hippocampus of 5xFAD;TfR^mu/hu^ mice compared to WT;TfR^mu/hu^ controls. 20 h p.i. emerged as a robust imaging time point since it allowed to detect differences between genotypes also in the hippocampus as a smaller and more challenging region compared to the frontal cortex. We also noticed spill-in from adjacent bone and endeavored to optimize our target regions by a data-driven approach using voxel-wise statistical parametric mapping. Here, PET-to-biodistribution correlation showed that elevated radiotracer uptake in whole brain of 5xFAD;TfR^mu/hu^ mice was mostly driven by voxels in the deeper layers of the frontal cortex, and good correlation between both read-outs was achieved, especially at 20 h p.i. This indicated a high accuracy of regional PET quantification when a safety margin is considered at the edge of the brain, and the overall high correlation validated the reliability of PET imaging at that time point. hTfR-related signal represented 80% of the brain signal in WT;TfR^mu/hu^ mice with low TREM2 expression levels. Given that hTfR expression is identical in 5xFAD;TfR^mu/hu^ 41, it can be inferred that the significant increase in the PET signal in 5xFAD;TfR^mu/hu^ cortex depends on TREM2. Thus, we speculate that biodistribution rather reflects hTfR-driven tracer uptake, whereas PET uncovers the activated DAM-driven brain retention in TREM2-enriched regions. Lacking biodistribution differences between genotypes at the group level appeared reasonable, since regional analysis by PET allows a local assessment of tracer uptake alterations, whereas biodistribution accounts for overall tracer uptake in the brain, including basal activity levels. It is also noteworthy, that the fraction of TREM2 binding in the cortex of 5xFAD;TfR^mu/hu^ mice is comparable in 5xFAD mice. Hence, ATV technology increases the retention of antibodies in the brain via hTfR binding, strongly increasing TREM2 signaling 41, but does not increase the relative proportion of TREM2 binding. The results of our small animal TREM2 PET investigation were confirmed by *ex vivo* autoradiography showing distinct differences in cortex-to-cerebellum ratios between 5xFAD;TfR^mu/hu^ mice and WT;TfR^mu/hu^ controls. Regional TREM2 PET sensitivity will increase in a human setting where the brain signal is less affected by adjacent structures due to higher PET resolution relative to the brain size.

Ultimately, we sought to prove the cellular selectivity of the novel TREM2 PET agents. In this regard, scRadiotracing has been shown to be a powerful methodology to disentangle cellular sources of PET signals over a variety of different cell types 46, 47. This methodology was already applied for radiotracers, which are taken up and retained within the cell, such as [^18^F]FDG and [^18^F]GE-180. We showed that this also applies to ligands of membrane-bound targets on the cell surface. At this point, we cannot distinguish if the measured activity comes from the surface-bound tracer or from the internalized radioligand-receptor complex. Therefore, we showed high cellular specificity of the ATV:4D9 tracer targeting microglia. Microglial enrichment correlated well with the TREM2 PET signal in aged App^SAA^;TfR^mu/hu^ mice. Thus, our cell sorting experiments provide strong evidence that TREM2 PET using ATV:4D9 is a powerful tool to monitor TREM2 on microglia.

While our preclinical data with ATV:4D9 are promising, translation into the clinic warrants a radiotracer which targets human TREM2. Hence, we prepared a radiotracer using the 14D3 antibody, which binds hTREM2 specifically (Figure S11). *In vitro* autoradiography experiments employing AD brain sections and [^64^Cu]Cu‑NODAGA-14D3 revealed a distinct detection primarily concentrated in the cortex, which overlaps with TREM2-expressing microglia and typical Aβ plaque and phosphorylated Tau protein deposits in the cortex. This is consistent with clustering of TREM2-positive microglia around amyloid plaques in both mice and humans 24, 32, 59.

Attempts to detect TREM2 via imaging in the CNS using PET have been reported previously using a bispecific antibody construct 8. ^124^I-labeled mAb1729 was used to target TREM2 and scFv8D3 for TfR-mediated transcytosis. The radiotracer showed good brain uptake and could detect TREM2 *ex vivo*. However, it failed to detect TREM2 *in vivo*. This was explained by high blood retention and possibly poor target binding due to TREM2 shedding and low TREM2 affinity. In our study, [^64^Cu]Cu‑NODAGA-ATV:4D9 revealed lower blood retention which could be attributed to a smaller antibody format compared to the mAb1729-scFv8D3 construct or target-mediated drug disposition. TREM2 agonist activity was not impaired compared to the native antibody (0.03 nM for ATV:4D9) 41, indicating preserved affinity and epitope upon radiotracer labeling.

The application of TREM2 PET imaging could extend beyond Alzheimer's disease to other brain conditions, notably leukodystrophies that are linked to microglial activation. The critical function of TREM2 is highlighted by the homozygous loss-of-function mutation that results in Nasu-Hakola disease, which is characterized by central microglial pathology 60, 61. Leukodystrophies resulting from lysosomal or peroxisomal deficiencies also significantly affect microglia, resulting in dysfunctional myelination and white matter maintenance 62, 63. It has been demonstrated that demyelination and the death of oligodendrocytes result in astrogliosis, microglial activation, and macrophage recruitment, with immune activation frequently occurring prior to white matter damage 64, 65. While FDG PET has been used to examine cerebral glucose metabolism alterations 66-68, TREM2 PET could provide a novel approach for evaluating leukodystrophies given the pivotal role of microglia in their pathogenesis. Furthermore, TREM2 PET may prove valuable in assessing traumatic brain injury, considering the correlation between microglial activation and the progression to chronic traumatic encephalopathy and other related disorders 69, 70.

Some limitations have to be considered within this study. 4D9 not only targets membrane-bound TREM2, but also soluble TREM2 (sTREM2) 12, 37, 71. It has to be clarified to what extent sTREM2 might trap the radiotracer, and if binding to diffusible sTREM2 facilitates clearance or deteriorates the spatial resolution of the PET images. In future, alternative TREM2 epitopes could be considered to optimize TREM2 as a radiotracer target. Our ongoing studies will concentrate on characterizing the specific role of TfR binding using PET and single cell Radiotracing 72. Furthermore, longitudinal TREM2 PET imaging will be important to investigate if the radiotracer is able to image alterations of microglial activation during disease progression.

## Conclusions

In conclusion, we present the first microglia-specific PET radiotracer, which proved suitable for imaging TREM2-associated activation of microglia in the CNS in a mouse model of AD. We leveraged ATV technology to improve the efficiency of tracer delivery across the BBB. To pave the way for a human application, we performed preliminary experiments with a radiotracer targeting human TREM2. Results obtained from immunohistochemistry and *in vitro* autoradiography on brain sections of AD patients provide a strong rationale for future translation of this research into clinical applications. TREM2 PET imaging has great potential to verify target engagement and to monitor therapy response in antibody-based TREM2 agonistic interventions.

## Supplementary Material

Supplementary figures and tables.

## Funding

This work was supported by the Deutsche Forschungsgemeinschaft (DFG, German Research Foundation) with individual applications (ID 495961210, BR 4580/3-1, LI 3533/1-1) to SL and MB and under Germany's Excellence Strategy within the framework of the Munich Cluster for Systems Neurology (EXC 2145 SyNergy - ID 390857198 to CH and MB), by the DFG research unit FOR2858 (ID 403161218 to MB) and a Koselleck Project HA1737/16-1 (to CH).

## Figures and Tables

**Table 1:** Tracers used for individual mouse lines.

Cohort	Mouse line	Tracer
1A	5xFAD	[^64^Cu]Cu‑NODAGA-4D9
1B	WT	[^64^Cu]Cu‑NODAGA-4D9
2A	5xFAD;TfR^mu/hu^	[^64^Cu]Cu‑NODAGA-ATV:4D9
2B	WT;TfR^mu/hu^	[^64^Cu]Cu‑NODAGA-ATV:4D9 [^64^Cu]Cu‑NODAGA-ATV:ISO
3	APP^SAA^;TfR^mu/hu^	[^64^Cu]Cu‑NODAGA-ATV:4D9

**Table 2:** ** Results of ^64^Cu-labeling.** RCY and RCP are given as mean (range), A_s_ as mean ± SD. The RCP was obtained from radio-TLC quality control measurements.

Tracer	RCY (%)	RCP (%)	A_s_ (MBq/µg)
[^64^Cu]Cu‑NODAGA-4D9	69.4 (60.0-85.7) (n = 5)	98.5 (95.8-99.5) (n = 4)	1.4 ± 0.3 (n = 4)
[^64^Cu]Cu‑NODAGA-ATV:4D9	67.1 (61.3-72.6) (n = 6)	97.3 (90.5-100) (n = 6)	1.0 ± 0.2 (n = 6)

RCY: radiochemical yield; RCP: radiochemical purity; A_s_: specific activity.

## Abbreviations

AD: Alzheimer's disease
ARG: autoradiography
ATV: antibody transport vehicle
BBB: blood-brain barrier
CBL: cerebellum
CNS: central nervous system
CT: computed tomography
CTX: cortex
DAM: disease-associated microglia
HIP: hippocampus
HPLC: high performance liquid chromatography
hTfR: human transferrin receptor
IHC: immunohistochemistry
MACS: magnetic cell separation
MRI: magnetic resonance imaging
PD: Parkinson's disease
PET: positron emission tomography
RCP: radiochemical purity
RCY: radiochemical yield
ROI: region of interest
SDS-PAGE: sodium dodecyl sulfate polyacrylamide gel electrophoresis
scRadiotracing: single cell Radiotracing
SEC: size exclusion chromatography
SPM: statistical parametric mapping
TLC: thin layer chromatography
TREM2: Triggering receptor expressed on myeloid cells 2
TSPO: 18 kDa translocator protein
VOI: volume of interest
