# Supplementary material for: PET imaging of microglia in Alzheimer's disease using copper-64 labeled TREM2 antibodies
Source: Theranostics. 2024 Sep 30;14(16):6319–36. doi: 10.7150/thno.97149 (PMC11488106; doi:10.7150/thno.97149)
Supplement: Supplementary file 1 — Supplementary figures and tables. [file thnov14p6319s1.pdf]

Supplement to:
PET imaging of microglia in Alzheimer's disease using copper-64 labeled TREM2 antibodies

Monireh Shojaei[1*], Rebecca Schaefer[1*], Kai Schlepckow[2], Lea H. Kunze[1], Felix L. Struebing[2,3], Bettina Brunner[2], Michael Willem[2], Laura M. Bartos[1], Astrid Feiten[2,4], Giovanna Palumbo[1], Thomas Arzberger[3,5], Peter Bartenstein[1,6], Gian Carlo Parico[7], Dan Xia[7], Kathryn M. Monroe[7], Christian Haass[2,4,6], Matthias Brendel[1,2,6*], Simon Lindner[1*]

[1] Department of Nuclear Medicine, University Hospital, LMU Munich, Munich, Germany

[2] German Center for Neurodegenerative Diseases (DZNE), Munich, Germany

[3] Center for Neuropathology and Prion Research, University Hospital, LMU Munich, Munich, Germany

[4] Metabolic Biochemistry, Biomedical Center (BMC), Faculty of Medicine, LMU Munich, Munich, Germany

[5] Department of Psychiatry and Psychotherapy, University Hospital, LMU Munich, Munich, Germany

[6] Munich Cluster of Systems Neurology (SyNergy), Munich, Germany

[7] Denali Therapeutics Inc, South San Francisco, CA, USA

*contributed equally

SUPPLEMENTAL METHODS

CHEMICALS

Chemicals were obtained from the following companies: CheMatech, Thermo Fischer Scientific macrocycle design technologies, Merck, Sigma Aldrich, VWR, and Advanced Biochemical Compounds (ABX). All chemicals were utilized directly without any further purification. [^{64}Cu]$CuCl_2$ was received from the Department of Preclinical Imaging and Radiopharmacy, University Hospital Tuebingen, Germany. 14D3 [1], 4D9 (Schlepckow *et al*, 2020 [2]), and ATV:4D9 antibody (Van Lengerich *et al*, 2023 [3]) were provided by DENALI Therapeutics, South San Francisco, United States, and German Center for Neurodegenerative Diseases (DZNE Munich).

p-SYK ASSAY

Quantification of p-SYK levels in HEK293 cells stably overexpressing TREM2 and DAP12 upon stimulation with either 4D9 or ATV:4D9 was determined using the AlphaLISA technology. The experiment was performed as described previously by Schlepckow *et al*. 2020 [2].

IN VITRO STABILITY

[^{64}Cu]Cu-NODAGA-ATV:4D9 was incubated in murine plasma for 48 h. After neck dislocation of the mouse, the blood was collected by cardiac puncture and transferred into an Eppendorf tube. The blood samples were centrifuged for 10 minutes at 2000 rpm (Mini spin centrifuge, Eppendorf), and blood cells were separated from the plasma, which was subsequently kept at -20 °C. The tracer (172.5 MBq) was incubated in 100 µL of murine plasma with gentle shaking (400 rpm) at 37 °C for 48 h. After 30 min, 180 min, 270 min, 19 h, 42 h and 48 h, 10 µL of the mixture was taken and injected into HPLC (Agilent Technologies, 1200 series, Phenomenex column, BioSep ™ 5 µm SEC-s 4000 500 Å LC Column 300 x 7.8 mm, with 0.1 M sodium phosphate buffer, pH 7.2, isocratic run, 1 mL/min, 20 min).

SDS-PAGE

Antibody integrity was assessed by SDS-PAGE under non-reducing conditions for unmodified antibodies 4D9 and ATV:4D9, modified antibodies NODAGA-4D9 and NODAGA-ATV:4D9, and labeled antibodies [^{64}Cu]Cu-NODAGA-4D9 and [^{64}Cu]Cu-NODAGA-ATV:4D9. Antibodies (2 µg in PBS) were incubated with SDS sample buffer (bio-rad, #1610747), loaded onto an 8% Bolt Bis-Tris Plus gel and run with MOPS buffer at 200 V for 35 min. Seeblue™ Plus2 Protein Ladder (Thermo Fisher Scientific) was used as a standard. Proteins were stained using Coomassie staining (SimplyBlue Safestain, Thermo Fisher Scientific) for one hour. The radioactive gels were exposed to a phosphor imaging plate for 30 minutes. The plates were scanned with a CR-Reader (CR35 BIO, Dürr Medical), and analyzed using Aida Image Analyzer software.

Analysis of antibody integrity and functionality of the NODAGA modification was performed *ex vivo* using plasma samples from mice 20 h p.i. treated with [^{64}Cu]Cu-NODAGA-4D9 (39.8 MBq, WT, n = 1, male) or [^{64}Cu]Cu-NODAGA-ATV:4D9 (42.3 MBq, WT;$TfR^{mu/hu}$, n = 1, male). Cardiac blood was collected in EDTA tubes (Sarstedt Microvette 100 K3E), centrifuged (3000 × g, 10 min) and the plasma was separated. Radioactivity measurements using a gamma counter were used to quantify tracer concentrations in the plasma. SDS-PAGE and autoradiography were conducted as previously described, except that 0.2 µg of antibody was loaded per well.

TRACER BENCHMARKING

%ID/g and SUVR values from PET studies utilizing a range of TSPO tracers were compiled. Ratios were calculated by comparing TSPO-rich tissues with reference tissues, and compared to ratios derived from [^{64}Cu]Cu-NODAGA-4D9 PET data of 5xFAD;TfR$^{mu/hu}$ and WT;TfR$^{mu/hu}$ 20 h p.i. in the frontal cortex.

IMMUNOHISTOCHEMISTRY

AD brain tissue was taken from an 83-year-old female patient with following characteristics: AD Braak stage VI, Aβ phase 5 according to Thal, cerebral amyloid angiopathy (especially in leptomeninx, occasionally in neocortex) stage 2 according to Thal, CERAD C + Lewy Body Disease (amygdala predominantly) + TDP43 stage 2 according to Josephs. The TREM2 genotype was unknown.

Immunohistochemistry was performed on paraffin sections using a Ventana BenchMark ULTRA (Roche). Primary antibodies were (a) mouse anti-beta-amyloid (17-24) (clone 4G8; diluted 1:5,000; Biolegend SIG-39220) and (b) mouse anti-hyperphosphorylated microtubule-associated protein tau (MAPT) (clone AT-8; diluted 1:400; ThermoFisher #MN1020). Pretreatment for antibody (a) was 80% formic acid for 15 min and antibody (b) boiling in CC1 buffer for 36 min. Diaminobenzidine/peroxidase-based detection system was UltraView. Stains were scanned with a Zeiss Axio Scan.Z1.

AUTORADIOGRAPHY OF HUMAN BRAIN SECTION

In vitro autoradiography of the human brain section was conducted as described for 5xFAD;TfR$^{mu/hu}$ and WT;TfR$^{mu/hu}$ mice (Methods, main manuscript).

SUPPLEMENTAL FIGURES

FIGURE S1 - ARSENAZO SPECTROPHOTOMETRIC ASSAY

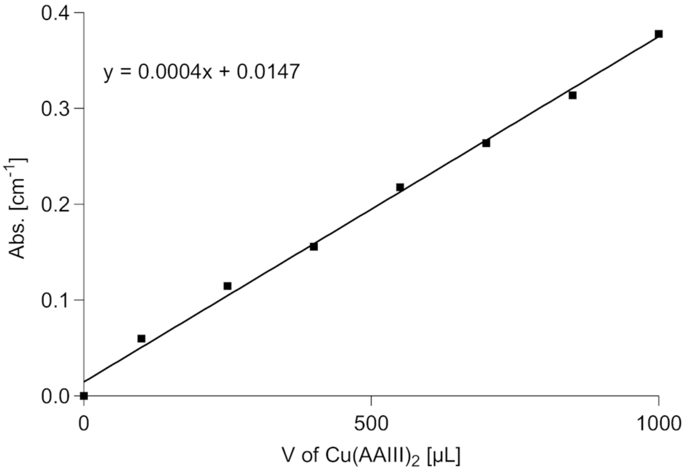

Validation of Lambert Beer’s law. The absorbance of different concentrations of $Cu(AAIII)_2$ in 0.15 M NH_4OAc, pH 7.0 was measured at 652 nm in a 1.0 mL quartz cuvette using a UV-Vis spectrophotometer. Linear regression, R^2 = 0.9957. Modified from [4].

FIGURE S2 - QUALITY CONTROL OF [^{64}Cu]Cu-NODAGA-ATV:4D9 AND [^{64}Cu]Cu-NODAGA-4D9

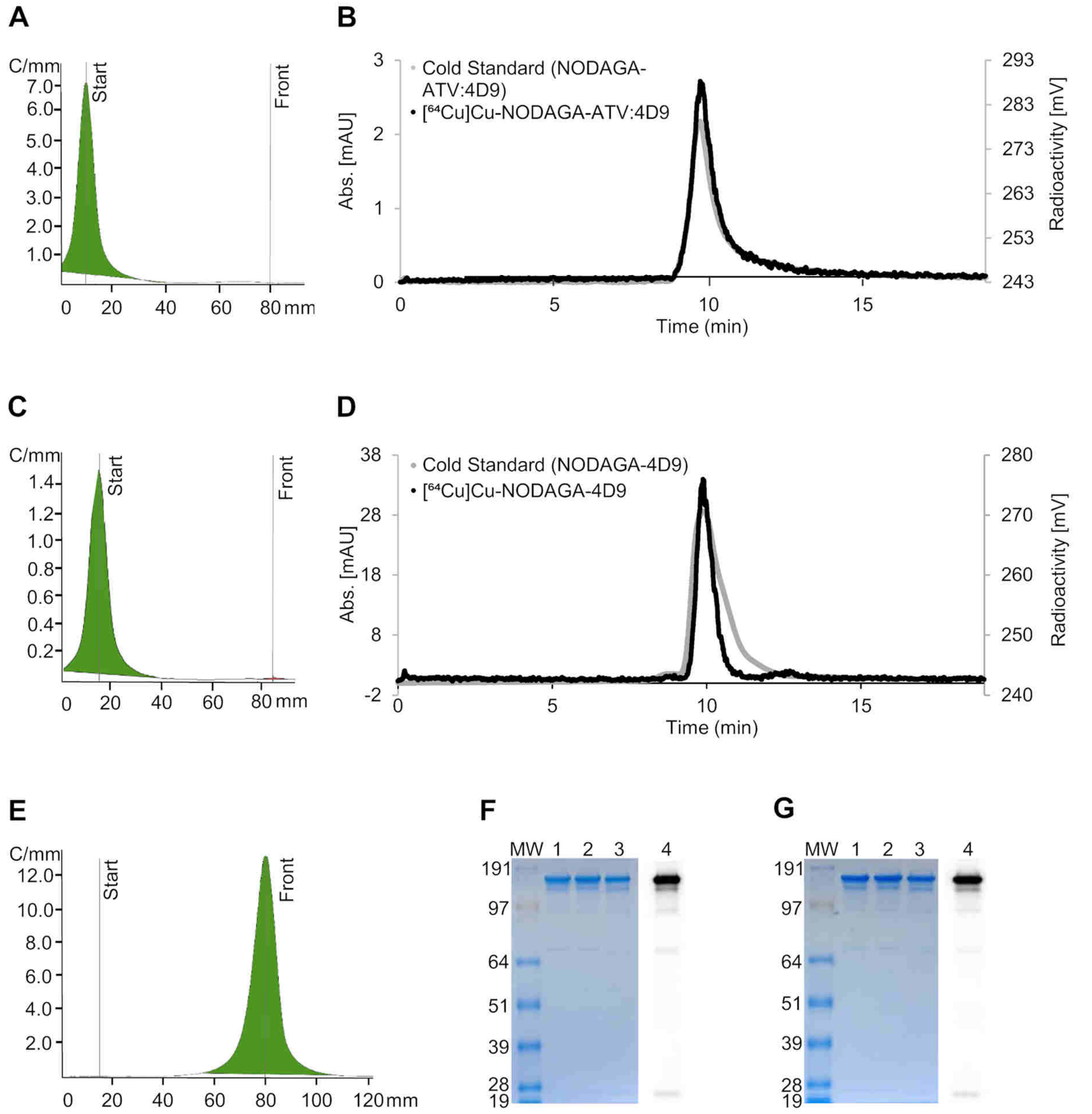

(A) Radio-TLC of [^{64}Cu]Cu-NODAGA-ATV:4D9 on ITLC-SG chromatography paper; R_f = 0.0. (B) HPLC chromatogram of [^{64}Cu]Cu-NODAGA-ATV:4D9; NODAGA-ATV:4D9 (R_t = 10.1 min, UV channel); [^{64}Cu]Cu-NODAGA-ATV:4D9 (R_t = 10.1 min, radio channel). (C) Radio-TLC of [^{64}Cu]Cu-NODAGA-4D9 on ITLC-SG chromatography paper; R_f = 0.0. (D) HPLC chromatogram of [^{64}Cu]Cu-NODAGA-4D9; NODAGA-4D9 (R_t = 10.3 min, UV channel); [^{64}Cu]Cu-NODAGA-4D9 (R_t = 10.3 min, radio channel). (E) Radio-TLC of [^{64}Cu]$CuCl_2$ on ITLC-SG chromatography paper; R_f = 1.0. (F) SDS-PAGE of ATV:4D9 (1), NODAGA-ATV:4D9 (2) and [^{64}Cu]Cu-NODAGA-ATV:4D9 (3) with autoradiography (4) of the SDS-PAGE gel. (G) SDS-PAGE of 4D9 (1), NODAGA-4D9 (2) and [^{64}Cu]Cu-NODAGA-4D9 (3) with autoradiography (4) of the SDS-PAGE gel.

FIGURE S3 - STABILITY OF [^{64}Cu]Cu-NODAGA-ATV:4D9

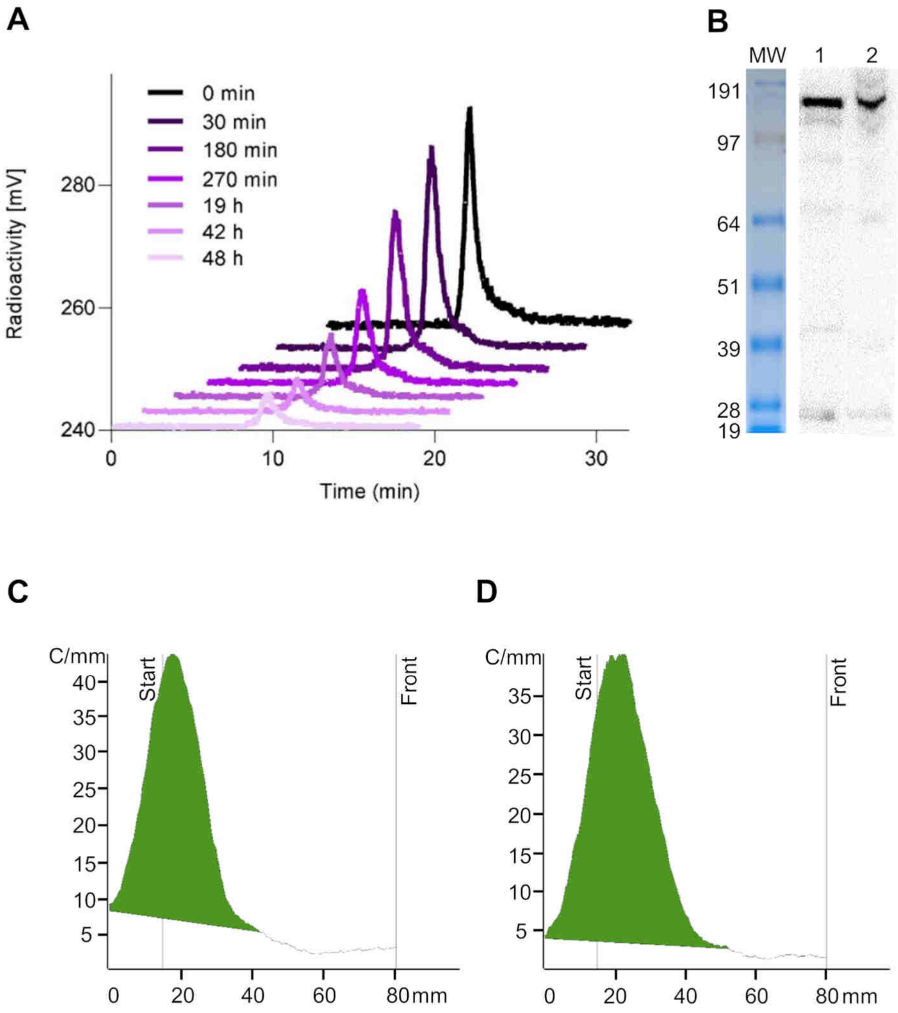

(A) *In vitro* stability of [^{64}Cu]Cu-NODAGA-ATV:4D9 in murine plasma over 48 h measured by SEC-HPLC (radioactivity channel). (B) Autoradiography of an SDS-PAGE gel loaded with plasma from a [^{64}Cu]Cu-NODAGA-4D9 (1) injected WT mouse and from a [^{64}Cu]Cu-NODAGA-ATV:4D9 (2) injected WT;TfR$^{mu/hu}$ mouse. (C) Radio-TLC of plasma from a [^{64}Cu]Cu-NODAGA-4D9 injected WT mouse on ITLC-SG chromatography paper, R_f (tracer) = 0.0-0.1. (D) Radio-TLC of plasma from a [^{64}Cu]Cu-NODAGA-ATV:4D9 injected WT;TfR$^{mu/hu}$ mouse on ITLC-SG chromatography paper, R_f (tracer) = 0.0-0.1.

FIGURE S4 - BRAIN BIODISTRIBUTION

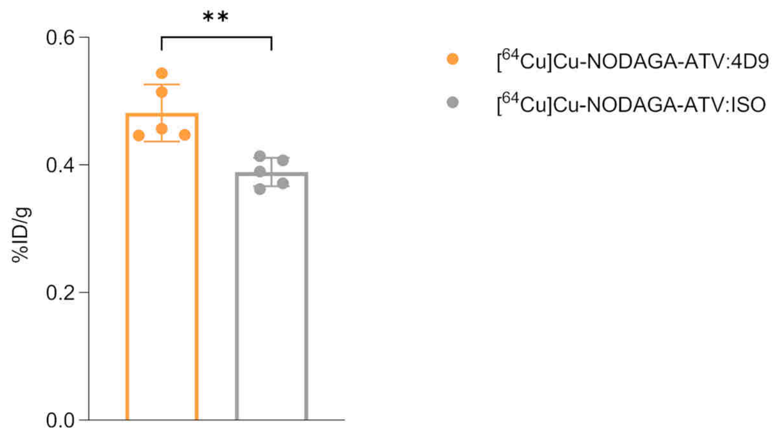

WT;TfR$^{mu/hu}$ mice were administered 14.9 ± 0.7 MBq (corresponding to 11.9 ± 0.6 µg per mouse) [^{64}Cu]Cu-NODAGA-ATV:ISO (n = 1, RCY = 85.6%, RCP = 100.0%, A_S = 1.3) in 150 µL phosphate buffer by intravenous injection through the tail vein. The relative brain uptake was determined after intracardial perfusion by biodistribution in WT;TfR$^{mu/hu}$ mice (n = 5 per group, female = 7, male = 3; 10-12 months) at 20 h p.i. Unpaired t-test, p = 0.0032 (**), mean ± SD.

FIGURE S5 - NON-DECAY-CORRECTED BRAIN BIODISTRIBUTION

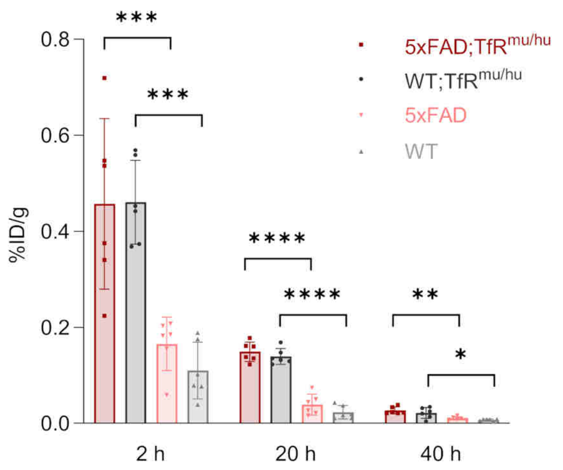

Non-decay-corrected brain uptake determined by biodistribution after intracardial perfusion in 5xFAD;TfR$^{mu/hu}$, 5xFAD, WT;TfR$^{mu/hu}$ and WT mice at 2 h, 20 h and 40 h p.i. One-way ANOVA/Tukey's multiple comparison test, $p \leq 0.05$ (*), $p \leq 0.01$ (**), $p \leq 0.001$ (***) and $p \leq 0.0001$ (****), mean ± SD.

FIGURE S6 - PET IMAGES 2 h AND 40 h p.i.

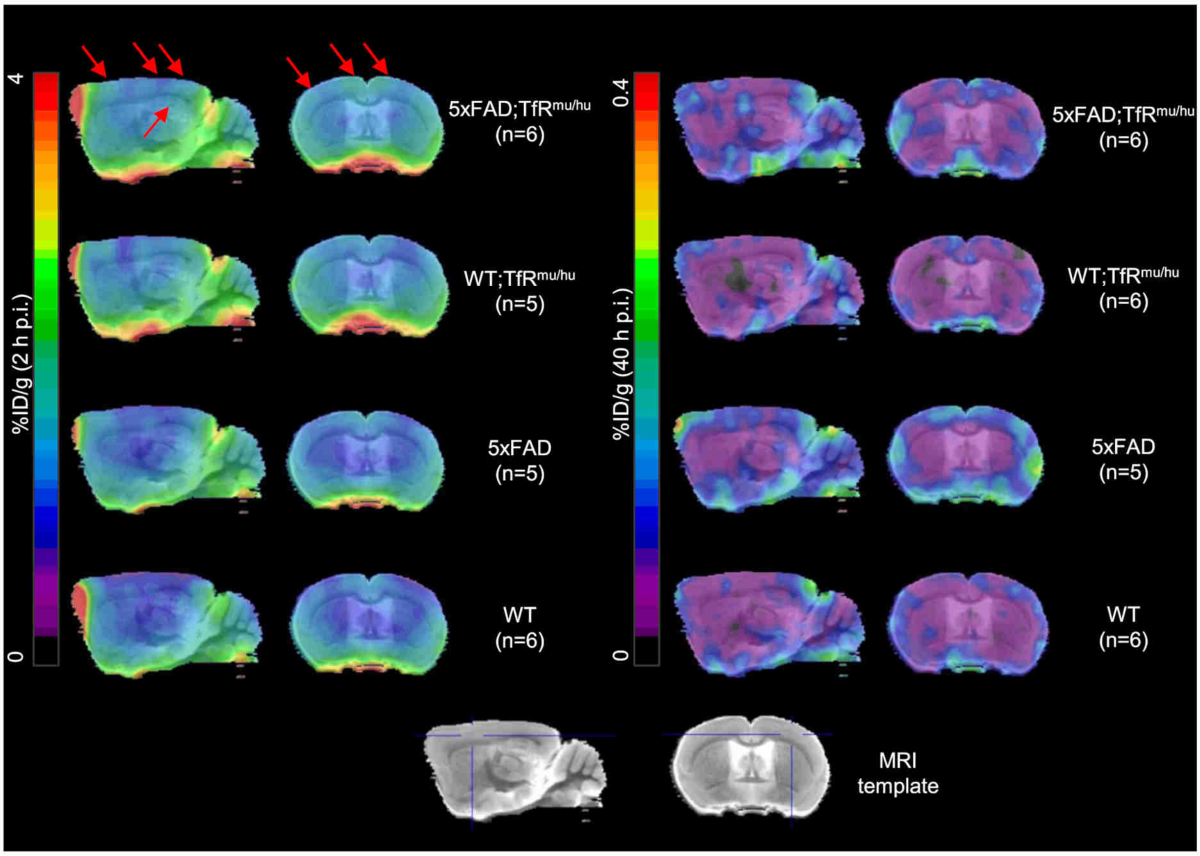

Tracer uptake in the brain of 5xFAD;TfR$^{mu/hu}$, WT;TfR$^{mu/hu}$, 5xFAD and WT mice at 2 h p.i. (left) and 40 h p.i. (right) in %ID/g.

FIGURE S7 - TREM2 PROTEIN LEVELS IN MOUSE BRAIN LYSATES

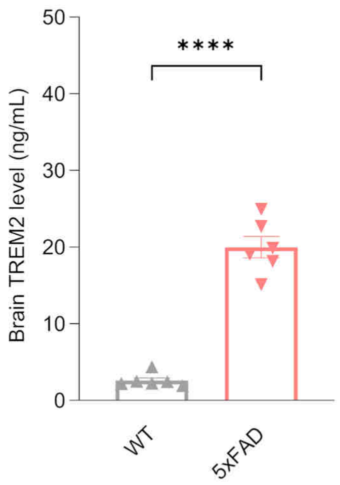

Brains were collected from 5xFAD and control animals around 6 months of age after cold PBS perfusion. Brain cortex was dissected out for homogenization using lysis buffer (Cell Signaling #9803) containing protease inhibitor cocktail (Roche #4693159001) and PhosSTOP (Roche #4906837001) as described previously [5]. After centrifugation, supernatants were transferred to new tubes for protein concentration and mouse TREM2 analysis. Mouse TREM2 levels were determined in diluted brain lysates (1:5) by an electrochemiluminescence-based assay using the Meso Scale Discovery Platform as described before [2, 5]. MSD values acquired on the MSD Sector Imager S600 reader were converted to absolute quantities of TREM2 by interpolating from a 4-parameter logistic curve fit to the mouse TREM2 standard using Graphpad Prism software and then normalized to the protein concentrations of each sample. Unpaired t-test, $p < 0.0001$ (****), mean ± SD.

FIGURE S8 - TRACER BENCHMARKING

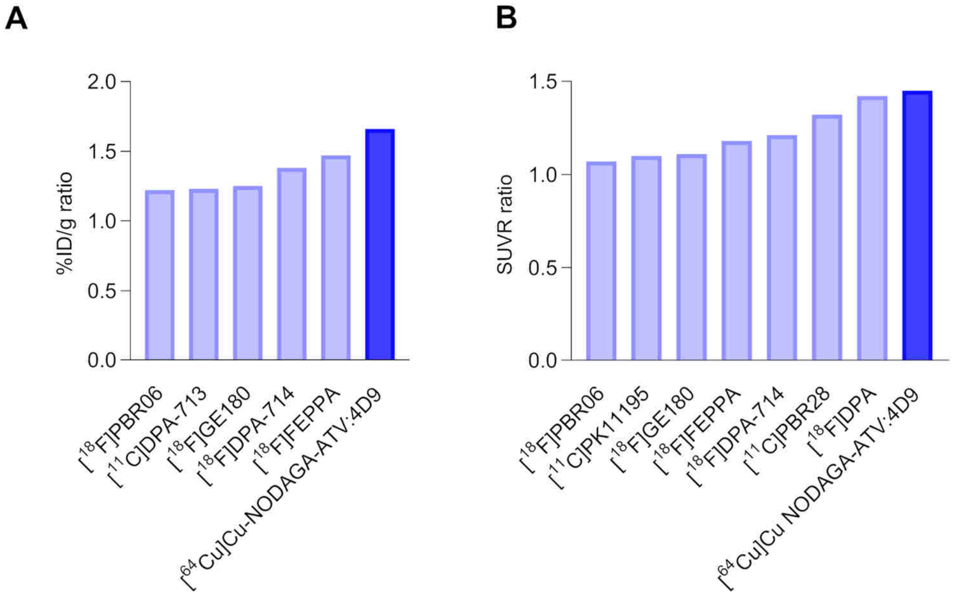

A benchmark comparison of multiple TSPO PET tracers vs [^{64}Cu]Cu-NODAGA-ATV:4D9. TSPO data derived from other PET studies (Tables S6 and S7) were used to calculate a ratio between target positive and reference tissue. Ratios are shown as (A) %ID/g and (B) SUVR.

FIGURE S9 - FLOW CYTOMETRY GATING STRATEGY

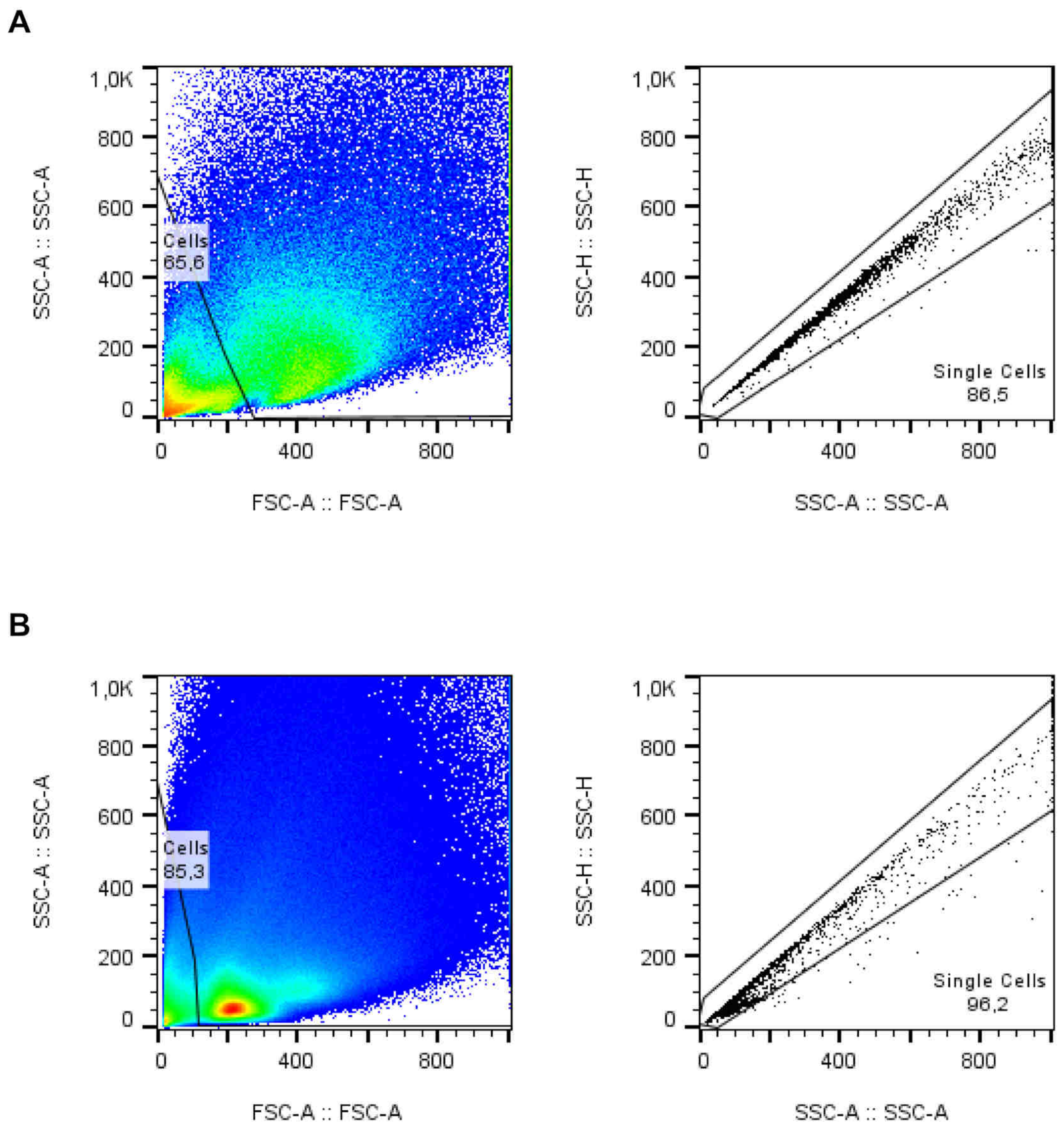

Flow cytometry gating strategy of the CD11b enriched fraction (A) and the CD11b depleted fraction (B). Left panels show pooled data from APPSAA;TfR$^{mu/hu}$ mice (n = 4) of total cells, right panels singlets considered for cell count and purity assessment.

FIGURE S10 - PET IMAGES OF APP^{SAA};TfR$^{mu/hu}$ vs WTx;TfR$^{mu/hu}$ 20 h p.i.

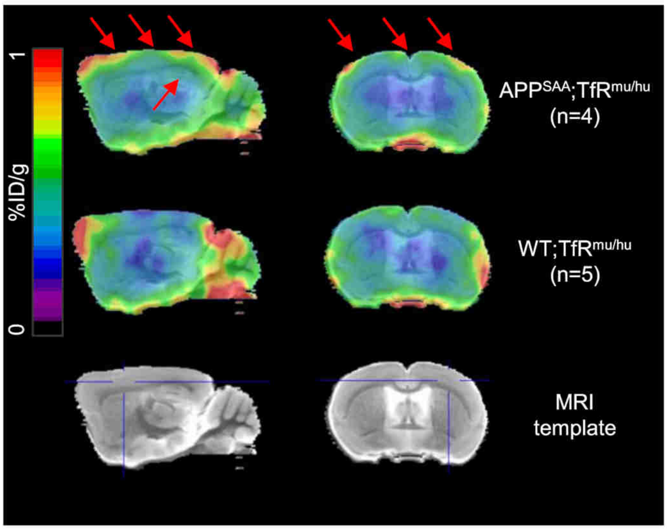

Tracer uptake in brains of APP^{SAA};TfR$^{mu/hu}$ 20 h p.i. in %ID/g. WT;TfR$^{mu/hu}$ images, derived from Fig. 3, are shown for comparison purposes.

FIGURE S11 - SPECIES SELECTIVITY OF 14D3 AND 4D9

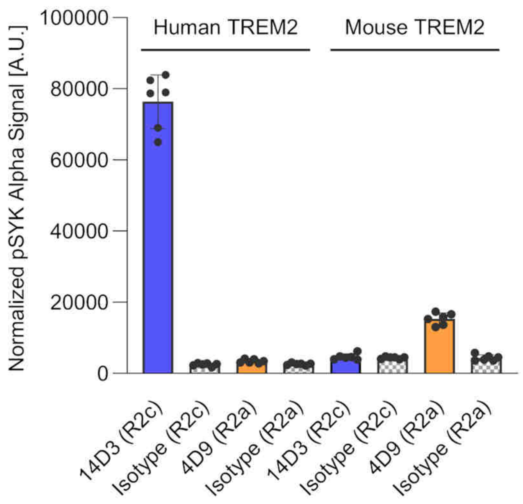

AlphaLISA mediated quantification of p-SYK levels (normalized to protein concentration) in HEK293 Flp-In cells stably overexpressing either human TREM2 and human DAP12, or mouse TREM2 and mouse DAP12 stimulated with 20 µg/µL hTREM2 14D3 (R2c), isotype control (R2c), mTREM2 4D9 (R2a) and isotype control (R2a). Data represent mean ± SD (n = 2 in triplicates).

FIGURE S12 - CO-LOCALIZATION OF IHC (Aβ AND TAU PROTEIN) AND ARG

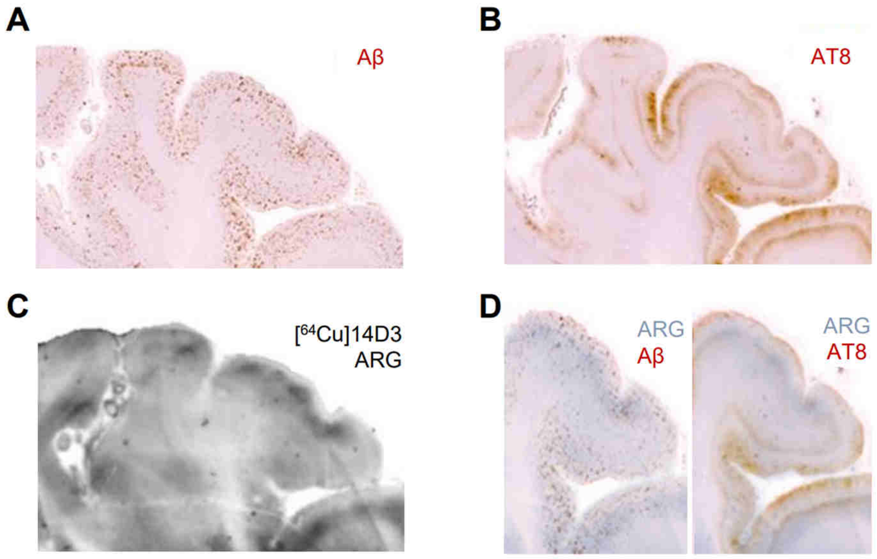

Immunohistochemistry of occipital brain sections derived from a patient with Alzheimer’s disease (AD) revealed cortical binding of (A**)** β-amyloid and (B) pTau. (C) *In vitro* autoradiography (ARG) of [^{64}Cu]Cu-NODAGA-14D3. (D) Aβ and pTau pathology were co-localized with tracer binding.

SUPPLEMENTAL TABLES

TABLE S1 BRAIN UPTAKE FROM BIODISTRIBUTION EXPERIMENTS

Decay-corrected data:

**%ID/g Mean ± SD**	**5xFAD;$TfR^{mu/hu}$**	**WT;$TfR^{mu/hu}$**	**5xFAD**	**WT**	**F statistics**
2 h p.i.	0.57 ± 0.20	0.56 ± 0.09	0.21 ± 0.07	0.15 ± 0.07	F (3, 20) = 17.63, P<0.0001
20 h p.i.	0.52 ± 0.06	0.46 ± 0.04	0.13 ± 0.07	0.10 ± 0.05	F (3, 20) = 75.59, P<0.0001
40 h p.i.	0.30 ± 0.07	0.23 ± 0.12	0.14 ± 0.04	0.10 ± 0.03	F (3, 20) = 9.350, P=0.0005

Non-decay-corrected data:

**%ID/g Mean ± SD**	**5xFAD;$TfR^{mu/hu}$**	**WT;$TfR^{mu/hu}$**	**5xFAD**	**WT**	**F statistics**
2 h p.i.	0.46 ± 0.18	0.46 ± 0.09	0.17 ± 0.06	0.11 ± 0.06	F (3, 20) = 18.29, P<0.0001
20 h p.i.	0.15 ± 0.02	0.14 ± 0.02	0.04 ± 0.02	0.02 ± 0.01	F (3, 20) = 76.57, P<0.0001
40 h p.i.	0.03 ± 0.01	0.02 ± 0.01	0.01 ± 0.00	0.01 ± 0.00	F (3, 20) = 10.43, P=0.0002

TABLE S2 BIODISTRIBUTION DATA 40 h p.i.

**%ID/g Mean ± SD**	**5xFAD;$TfR^{mu/hu}$**	**WT;$TfR^{mu/hu}$**	**5xFAD**	**WT**
Brain	0.30 ± 0.07	0.23 ± 0.12	0.14 ± 0.04	0.10 ± 0.03
Heart	0.87 ± 0.15	0.81 ± 0.33	1.47 ± 0.55	1.26 ± 0.57
Kidney	1.88 ± 0.31	1.67 ± 0.75	3.03 ± 1.57	2.64 ± 1.10
Pancreas	0.61 ± 0.15	0.54 ± 0.26	0.98 ± 0.48	0.75 ± 0.35
Spleen	4.48 ± 0.91	3.28 ± 1.61	5.63 ± 3.57	3.44 ± 1.53
Muscle	0.31± 0.09	0.24 ± 0.09	0.53 ± 0.17	0.47 ± 0.16
Bone	7.99 ± 2.51	10.15 ± 8.49	8.87 ± 3.10	6.54 ± 1.62
Lung	1.48 ± 0.29	1.30 ± 0.56	1.59 ± 1.21	2.01 ± 0.99
Liver	3.56 ± 0.33	2.69 ± 1.36	3.69 ± 1.23	4.76 ± 1.92
Blood	2.02 ± 0.76	1.19 ± 0.67	5.99 ± 2.79	3.47 ± 1.66

TABLE S3 UPTAKE IN CORTEX FROM PET ANALYSIS

**%ID/g Mean ± SD**	**5xFAD;$TfR^{mu/hu}$**	**WT;$TfR^{mu/hu}$**	**5xFAD**	**WT**	**F statistics**
2 h p.i.	2.25 ± 0.10	1.68 ± 0.28	1.45 ± 0.11	1.37 ± 0.21	F (3, 18) = 26.32, P<0.0001

20 h p.i.	0.90 ± 0.18	0.55 ± 0.05	0.48 ± 0.08	0.29 ± 0.11	F (3, 18) = 26.68, P<0.0001
40 h p.i.	0.11 ± 0.02	0.06 ± 0.03	0.11 ± 0.02	0.06 ± 0.02	F (3, 19) = 6.207, P=0.0040

TABLE S4 - UPTAKE IN HIPPOCAMPUS FROM PET ANALYSIS

**%ID/g Mean ± SD**	**5xFAD;TfR$^{mu/hu}$**	**WT;TfR$^{mu/hu}$**	**5xFAD**	**WT**	**F statistics**
2 h p.i.	1.45 ± 0.26	1.40 ± 0.29	1.31 ± 0.12	1.19 ± 0.30	F (3, 18) = 1.140, P=0.3597
20 h p.i.	0.55 ± 0.09	0.34 ± 0.08	0.30 ± 0.07	0.18 ± 0.07	F (3, 18) = 22.17, P<0.0001
40 h p.i.	0.06 ± 0.02	0.06 ± 0.05	0.07 ± 0.02	0.06 ± 0.03	F (3, 19) = 0.2938, P=0.8294

TABLE S5 - EFFECT SIZES EXPRESSED AS COHEN'S D FROM PET ANALYSIS

**Cohen´s d**	**Cortex**			**Hippocampus**		
	**2 h**	**20 h**	**40 h**	**2 h**	**20 h**	**40 h**
5xFAD;TfR$^{mu/hu}$ vs WT;TfR$^{mu/hu}$	2.68	2.62	1.75	0.16	2.50	-0.19
5xFAD;TfR$^{mu/hu}$ vs 5xFAD	7.44	2.98	0.18	0.68	3.30	-1.03

TABLE S6 - BENCHMARK COMPARISON IN %ID/g

**Tracer**	**Host**	**Overview pos vs ref**	**Region**	**%ID/g pos/ref/ratio**	**Ref**
[^{18}F]PBR06	mouse	APP$^{L/S}$ vs WT	CTX	4.07/3.33/1.22	[6]
[^{11}C]DPA-713	mouse	Neuroinflammation in ischemic stroke model (ipsilateral vs contralateral)	CTX	3.11/2.53/1.23	[7]
[^{18}F]GE180	mouse	Neuroinflammation in ischemic stroke model (ipsilateral vs contralateral)	CTX	4.26/3.42/1.25	[7]
[^{18}F]DPA-714	mouse	3xTg-AD vs WT	whole brain	1.35/0.98/1.38	[8]
[^{18}F]FEPPA	mouse	WT; Neuroinflammation induced by LPS vs saline	CTX	1.35/0.98/1.38	[9]
[^{64}Cu]Cu-NODAGA-ATV:4D9	mouse	5xFAD;TfR$^{mu/hu}$ vs WT;TfR$^{mu/hu}$	CTX	0.90/0.55/1.64	

TABLE S7 - BENCHMARK COMPARISON IN SUVR

Tracer	Host	Overview pos vs ref	Region	SUVR ref region	SUVR pos/ref/ratio	Ref
[^{18}F]PBR06	human	PD vs healthy	putamen	global brain	1.07/1.00/1.07	[10]
[^{18}F]PBR06	human	AD vs healthy	hippocampus	cerebellar cortex	1.07/0.97/1.10	[11]
[^{18}F]GE180	mouse	PS2APP vs WT	forebrain	white matter	0.71/0.64/1.11	[12]
[^{18}F]FEPPA	rat	Neuroinflammation induced by exposition to fine particulate matter (PM2.5) vs sterile filter	retrosplenial cortex	cerebellum	0.98/0.83/1.18	[13]
[^{18}F]DPA-714	mouse	APP/PS1-21 vs WT	CTX	cerebellum	0.88/0.73/1.21	[14]
[^{11}C]PBR28	mouse	APP/PS1-21 vs WT	CTX	cerebellum	0.96/0.73/1.32	[14]
[^{18}F]DPA	mouse	APP/PS1-21 vs WT	CTX	cerebellum	1.05/0.74/1.42	[14]
[^{64}Cu]Cu-NODAGA-ATV:4D9	mouse	5xFAD;TfR$^{mu/hu}$ vs WT;TfR$^{mu/hu}$	CTX	cerebellum	1.53/1.06/1.45	

TABLE S8 - UPTAKE IN CORTEX FROM SPM ANALYSIS

%ID/g Mean ± SD	5xFAD;TfR$^{mu/hu}$	WT;TfR$^{mu/hu}$	5xFAD	WT	F statistics
2 h p.i.	2.26 ± 0.22	1.80 ± 0.36	1.47 ± 0.07	1.50 ± 0.26	F (3, 15) = 10.24, P=0.0006
20 h p.i.	0.77 ± 0.14	0.52 ± 0.06	0.42 ± 0.08	0.27 ± 0.11	F (3, 18) = 24.33, P<0.0001
40 h p.i.	0.09 ± 0.02	0.06 ± 0.03	0.09 ± 0.02	0.06 ± 0.02	F (3, 19) = 3.719, P=0.0294

TABLE S9 - EFFECT SIZES EXPRESSED AS COHEN’S D FROM SPM ANALYSIS

Cohen´s d	Cortex		
	2 h	20 h	40 h
5xFAD;TfR$^{mu/hu}$ vs WT;TfR$^{mu/hu}$	1.57	2.39	1.35
5xFAD;TfR$^{mu/hu}$ vs 5xFAD	4.89	3.14	-0.03

REFERENCES

1. Haass C, Kleinberger G, Schlepckow K. TREM2 cleavage modulators and uses thereof. WO2018015573A2; 2018.
2. Schlepckow K, Monroe KM, Kleinberger G, Cantuti-Castelvetri L, Parhizkar S, Xia D, et al. Enhancing protective microglial activities with a dual function TREM2 antibody to the stalk region. EMBO Mol Med. 2020; 12: e11227.
3. van Lengerich B, Zhan L, Xia D, Chan D, Joy D, Park JI, et al. A TREM2-activating antibody with a blood–brain barrier transport vehicle enhances microglial metabolism in Alzheimer's disease models. Nat Neurosci. 2023; 26: 416-29.
4. Shojaei M, Zhou Q, Palumbo G, Schaefer R, Kaskinoro J, Vehmaan-Kreula P, et al. Development and Preclinical Evaluation of a Copper-64-Labeled Antibody Targeting Glycine-Alanine Dipeptides for PET Imaging of C9orf72-Associated Amyotrophic Lateral Sclerosis/Frontotemporal Dementia. ACS Pharmacol Transl Sci. 2024; 7: 1404-14.
5. Xia D, Lianoglou S, Sandmann T, Calvert M, Suh JH, Thomsen E, et al. Novel App knock-in mouse model shows key features of amyloid pathology and reveals profound metabolic dysregulation of microglia. Mol Neurodegener. 2022; 17: 41.
6. James ML, Belichenko NP, Nguyen T-VV, Andrews LE, Ding Z, Liu H, et al. PET Imaging of Translocator Protein (18 kDa) in a Mouse Model of Alzheimer's Disease Using *N*-(2,5-Dimethoxybenzyl)-2-^{18}F-Fluoro-*N*-(2-Phenoxyphenyl)Acetamide. J Nucl Med. 2015; 56: 311-6.
7. Chaney A, Cropper HC, Johnson EM, Lechtenberg KJ, Peterson TC, Stevens MY, et al. ^{11}C-DPA-713 Versus ^{18}F-GE-180: A Preclinical Comparison of Translocator Protein 18 kDa PET Tracers to Visualize Acute and Chronic Neuroinflammation in a Mouse Model of Ischemic Stroke. J Nucl Med. 2019; 60: 122-8.
8. Liu Y, Xu Y, Li M, Pan D, Li Y, Wang Y, et al. Multi-target PET evaluation in APP/PS1/tau mouse model of Alzheimer's disease. Neurosci Lett. 2020; 728: 134938.
9. Vignal N, Cisternino S, Rizzo-Padoin N, San C, Hontonnou F, Gelé T, et al. [^{18}F]FEPPA a TSPO Radioligand: Optimized Radiosynthesis and Evaluation as a PET Radiotracer for Brain Inflammation in a Peripheral LPS-Injected Mouse Model. Molecules. 2018; 23: 1375.
10. Liu S-Y, Qiao H-W, Song T-B, Liu X-L, Yao Y-X, Zhao C-S, et al. Brain microglia activation and peripheral adaptive immunity in Parkinson's disease: a multimodal PET study. J Neuroinflammation. 2022; 19: 209.
11. Wang Q, Chen G, Schindler SE, Christensen J, McKay NS, Liu J, et al. Baseline Microglial Activation Correlates With Brain Amyloidosis and Longitudinal Cognitive Decline in Alzheimer Disease. Neurol Neuroimmunol Neuroinflamm. 2022; 9: e1152.
12. Brendel M, Kleinberger G, Probst F, Jaworska A, Overhoff F, Blume T, et al. Increase of TREM2 during Aging of an Alzheimer's Disease Mouse Model Is Paralleled by Microglial Activation and Amyloidosis. Front Aging Neurosci. 2017; 9: 1-13.
13. Cheng M-F, Cheng T-J, Guo YL, Chiu C-H, Wu H-M, Yen R-F, et al. Neuroinflammation in Low-Level PM2.5-Exposed Rats Illustrated by PET via an Improved Automated Produced [^{18}F]FEPPA: A Feasibility Study. Mol Imaging. 2022; 2022: 1076444.
14. López-Picón FR, Keller T, Bocancea D, Helin JS, Krzyczmonik A, Helin S, et al. Direct Comparison of [^{18}F]F-DPA with [^{18}F]DPA-714 and [^{11}C]PBR28 for Neuroinflammation Imaging in the same Alzheimer's Disease Model Mice and Healthy Controls. Mol Imaging Biol. 2022; 24: 157-66.
